# Computer-assisted construction of Ramanujan–Sato series for 1 over $\pi$

**DOI:** 10.1007/s11139-026-01352-2

**Published:** 2026-02-26

**Authors:** Ralf Hemmecke, Peter Paule, Cristian-Silviu Radu

**Affiliations:** 1 Research Institute for Symbolic Computation (RISC), Johannes Kepler University, 4040 Linz, Austria; 2 Center for Applied Mathematics, Tianjin University, Tianjin, 300072 China

**Keywords:** Modular forms and functions, Holonomic differential equations, Ramanujan–Sato series for 1 over $\pi$, MultiSamba algorithm, Primary 05A30, 11F03, 68W30, Secondary 11F33, 11P83

## Abstract

Referring to ideas of Sato and Yang in (Math Z 246:1–19, 2004) described a construction of series for 1 over $\pi$ starting with a pair (*g*, *h*), where *g* is a modular form of weight 2 and *h* is a modular function; i.e., a modular form of weight zero. In this article we present an algorithmic version, called “Sato construction”. Series for $1/\pi$ obtained this way will be called “Ramanujan–Sato” series. Famous series fit into this definition, for instance, Ramanujan’s series used by Gosper and the series used by the Chudnovsky brothers for computing millions of digits of $\pi$. We show that these series are induced by members of infinite families of Sato triples $(N, \gamma _N, \tau _N)$ where $N>1$ is an integer and $\gamma _N$ a $2\times 2$ matrix satisfying $\gamma _N \tau _N=N \tau _N$ for $\tau _N$ being an element from the upper half of the complex plane. In addition to procedures for guessing and proving from the holonomic toolbox together with the algorithm “ModFormDE”, as described in Paule and Radu in Int J Number Theory (17:713–759, 2021), a central role is played by the algorithm “MultiSamba”, an extension of Samba (“subalgebra module basis algorithm”) originating from Radu in (J Symb Comput 68:225–253, 2015) and Hemmecke in (J Symb Comput 84:14–24, 2018). With the help of MultiSamba one can find and prove evaluations of modular functions, at imaginary quadratic points, in terms of nested algebraic expressions. As a consequence, all the series for $1/\pi$ constructed with the help of MultiSamba are proven completely in a rigorous non-numerical manner.

## Introduction

In his famous early paper [23], Ramanujan presented seventeen series as different representations for 1 over $\pi$. Laying dormant for more than seventy years, this part of Ramanujan’s work was brought back to the attention of a wider mathematical (and non-mathematical, e.g., [1] or  [5]) audience by Bill Gosper and, around the same time, by Jon and Peter Borwein [4].

Both Gosper and the Borweins were after efficient methods to compute digits of $\pi$: the Borweins by generalizing methods tracing back to Gauß’ arithmetic–geometric mean (AGM) algorithm, Gosper by using the last series listed by Ramanujan  [23, id. (44)] which is,

1 $$\begin{aligned} \frac{1}{\pi } = \frac{\sqrt{8}}{9801}\cdot \sum _{n=0}^\infty (26390 n+1103) \frac{(4n)!}{n!^4} \left( \frac{1}{396^4} \right) ^n. \end{aligned}$$

Based on this series, Gosper computed a continued fraction approximation to $\pi$ (his primary project goal) which converted to a decimal expansion gave more than 17 million digits of $\pi$, a world record at that time. Comparing this to the beginning of the 18th century when about 100 digits of $\pi$ were known, Askey commented [1, p. 895], “Part of the improvement comes from larger and faster machines, but much more comes from increased mathematical knowledge. Part of this is specific to certain numbers, and part is general and of wide applicability. All of it is very interesting.”

Indeed, the accomplishments of Gosper and the Borweins stimulated an avalanche of related developments; see, for example, the survey [2] by Baruah, Berndt, and Chan, the work by Chan and Cooper [8] presenting 186 series for $1/\pi$, Cooper’s beautiful and comprehensive monograph [12], or Zudilin’s article [32] entitled “Ramanujan-type formulae for $1/\pi$: A second wind?”

With the present article it is our hope to bring in new “algorithmic wind” into this area. One ingredient is based on holonomic methods: “ModFormDE”, an algorithm for proving conjectured linear differential equations satisfied by modular forms. The coefficients of such differential equations are polynomials in a given modular function. Details are given in [20] and [21].

The second ingredient, described in this article, is an algorithmic construction of Ramanujan–Sato series in which the algorithm “MultiSamba” is used as an essential tool. With the help of MultiSamba one can find and prove evaluations of modular functions, at imaginary quadratic points, in terms of nested algebraic expressions.

Some words on the mathematical background of Ramanujan–Sato series are in place. As an application of his work on differential equations for modular forms, Yifan Yang [31] referred to a construction of series for 1 over $\pi$ used by Takeshi Sato. Apart from the abstract [24], to our knowledge there is no further publication by Sato providing more details.

In the literature one finds various notions of “Ramanujan–Sato” series. In this article, the term *Ramanujan–Sato series* is reserved for a series of the type as on the right side of (13) which, in addition, can be obtained via the Sato construction as described in Sect. 3.

### Remark 1.1

According to Heng Huat Chan [7], the notion “Ramanujan–Sato type series” appeared the first time in the title of [9]. The series for 1 over $\pi$ presented there were motivated by series discovered by Sato, but were derived differently from the Sato construction.

The centerpiece of this article is the detailed algorithmic specification of the Sato construction in Sect. 3. We present a variety of examples to demonstrate the flexibility of our method. Concerning the series for $1/\pi$ which were derived using MultiSamba, we want to stress the following point: all the constants involved, i.e., the singular values of modular functions which find representations in terms of nested radicals, are fully proven.

The structure of this article is as follows.

Section 2 introduces all the basic notions used.

Section 3 presents Steps 0 to 8 of our algorithmization of the Sato construction described by Yang. All the steps are illustrated with the successive assembly of Ramanujan’s series (1) as a concrete running example. As already mentioned, the derivation using MultiSamba gives a complete proof of (1).

Section 4 presents an infinite family of Sato triples. Each such triple, in combination with the modular form setting used to obtain (1), gives a series for $1/\pi$ as the result of the Sato construction. Ramanujan’s (1) is a member of this family. Another family member is $\phi (m)$, a sum (85) which in the limit $m\rightarrow \infty$ gives $1/\pi$ and which adds 16 correct digits with each additional summand. This infinite Sato family can be viewed as an algorithmic counterpart to a general theorem by the Borweins [4, Eq. (5.5.16)]; see Theorem 4.4 below. Hemmecke’s implementation of our method in the computer algebra system FriCAS [14] derives the series (1) and other members from the infinite “Ramanujan-Gosper family” at the push of a button.

Section 5 presents two infinite families of Sato triples which induce series for $1/\pi$ as the result of the Sato construction. A prominent member of family 1 is the Chudnovsky series (87) which adds 14 correct digits with each additional summand, and which has been used until today for world-record computations of the digits of $\pi$; see [27]. The construction of series for $1/\pi$ from these families can be viewed as an algorithmic counterpart to a general theorem by the Chudnovskys [10, Eq. (1.4)], Theorem 5.4 below, which in its original formulation covers family 1 explicitly. Using Hemmecke’s implementation of MultiSamba in FriCAS derives the series (87) and other members from the infinite “Chudnovsky families 1 and 2” at the push of a button.

Section 6 presents again two infinite families of Sato triples inducing series for $1/\pi$. These series in a more direct way relate to the original work of Sato or, more precisely, to the work of Heng Huat Chan and collaborators [9] inspired by Sato. The underlying modular form setting is borrowed from work of Frits Beukers in connection with Apéry numbers. The construction of series for $1/\pi$ from the first Apéry–Beukers–Chan family can be viewed as an algorithmic counterpart to a general theorem by Heng Huat Chan and Helen Verrill [18, Eq. (5.1)]; see Theorem (6.3) below.

Section 7 is devoted to a description of our main computational engine, the algorithm MultiSamba to derive and prove algebraic relations between modular functions. When explaining the functionality of the algorithm MultiSamba, which has a sophisticated polynomial reduction procedure as its main ingredient, we restrict to the presentation of illustrating examples.

Sections 8 and 9 deal with those tasks of the algorithmic Sato construction for which MultiSamba is responsible. Step 0 of the construction is the choice of a pair (*g*, *h*), where *g* is a modular form of weight 2 and *h* is a modular function; i.e., a modular form of weight zero. The series ingredient of the Sato construction is a local expansion of the form,

2 $$\begin{aligned} g(\tau )=\sum _{n\ge 0} c(n) h(\tau )^n\, \,\text { for all }\, \tau \in \mathbb H\,\text { with }\, \Im (\tau ) \,\text { sufficiently large}. \end{aligned}$$

The evaluation of $t_N:=h(\tau _N)$ with $\tau _N$ an imaginary quadratic point is of special interest. Section 8 describes how an algebraic expression for $t_N$, ideally in the form of nested radicals, can be derived and proven algorithmically. Section 9 is devoted to a similar task of obtaining algebraic expressions for $p_1(t_N)$ and $p_2(t_N)$ where the $p_j$ are algebraic functions defined using *g*.

Sections 10 and 11 deal with algorithmic aspects related to (2); explanations as a concrete example use the pair (*g*, *h*) which makes the starting point for the construction of (1). Section 10 describes the algorithmic discovery and proving of local expansions (2) using the holonomic toolbox as described in detail in [21]. Section 11 describes, again for this concrete case, how a bound $L>0$ is derived such that for all $\tau$ with $\Im (\tau )>L$ the local expansion (2) holds.

In Sect. 12, for the sake of completeness, we prove the fact stated in Lemma 3.14; namely, that the modularity of the modular forms *g* and *h*, used in the construction of (1), indeed extends from $\Gamma (2)$ to a bigger group.

We conclude this Introduction with a remark on software: the RISC packages used, Mallinger’s package GeneratingFunctions written in Mathematica and Hemmecke’s package QEta written in FriCAS, are freely available at https://caa.risc.jku.at/software.

## Basic notions

In this section we introduce basic notions used throughout the paper.

For certain hypergeometric series arising in the text some readers could prefer standard ${\phantom {A}}_pF_q$-notation. To facilitate translation we use rising factorials,

$$ (a)_n:=a(a+1)\dots (a+n-1), n\ge 1, \,\text { and }\, (a)_0:=1. $$

By $\tau$ we denote an element of the upper half of the complex plane; i.e., $\tau \in \mathbb H=\{\tau \in \mathbb C: \Im (\tau )>0\}$. Moreover,

$$\begin{aligned} q:=\exp (2 \pi i \tau ), \,\text { and }\, q_w:=\exp (2 \pi i \tau /w) \,\text { for }\, w\in \mathbb Z_{\ge 1}. \end{aligned}$$

It will be convenient to introduce an additional short hand for the general Fourier variable if *w* is clear from the context,

$$ x:=q_w=\exp (2 \pi i \tau /w). $$

Throughout, $\Gamma$ will be a congruence subgroup of $\textrm{SL}_2(\mathbb {Z})$, or a subgroup of $\textrm{GL}_2^{+}(\mathbb Q)$ which in our context will be constructed as an extension of a congruence subgroup. The action of $\Gamma$ on elements from $\mathbb H\cup \mathbb Q\cup \{\infty \}$ is as usual,

$$ \left( \begin{matrix} a& b\\ c& d \end{matrix} \right) \tau := \frac{a \tau + b}{c \tau +d}. $$

For any complex function *f* on $\mathbb H$ and $k\in \frac{1}{2} \mathbb Z$ the slash operator $\mid _k$ for $$\gamma =\left( {\begin{smallmatrix} a& b\\ c& d \end{smallmatrix}} \right) \in \Gamma $$ is defined by

$$ (f\mid _k \gamma )(\tau ):= \det (\gamma )^{k/2} (c \tau +d)^{-k} f(\gamma \tau ). $$

To gain extra flexibility we allow for a character $\chi$ as a possible extra factor.

### Definition 2.1

Let *f* be a complex function on $\mathbb H$. We call *f* a modular form with weight $k\in \frac{1}{2} \mathbb Z$ and character $\chi$, if it satisfies the following three conditions:    (1) *f* is meromorphic on $\mathbb H$;    (2) $(f\mid _k \gamma )(\tau ) = \chi (\gamma ) f(\tau )$ for any $\gamma \in \Gamma$;    (3) *f* is meromorphic at each cusp point of $\Gamma$.

The set of all such modular forms is denoted by $M_k(\Gamma ; \chi )$ which is a vector space over $\mathbb C$. If $\chi (\gamma )=1$ for all $\gamma \in \Gamma$ we use the short hand,

$$ M_k(\Gamma ):=M_k(\Gamma , 1). $$

If, in addition, $k=0$ the elements of $M_0(\Gamma )$ form the field of meromorphic modular functions for $\Gamma$.

For $f\in M_k(\Gamma ; \chi )$ condition (3) of Definition 2.1 implies at the cusp $\infty$ a representation of the form

$$ f(\tau )=\sum _{n\ge m} a_n \exp (2 \pi i \tau /w)^n \,\,\,\text { for all }\, \tau \,\text { with }\,\Im (\tau ) \,\text { sufficiently large,} $$

and with $w\in \mathbb Z_{\ge 1}$ fixed. If $a_m\ne 0$ then ${{\,\textrm{ord}\,}}_\infty (f):= m$ is the (vanishing) order of *f* at infinity. It will be convenient to define

$$ \tilde{f}(z):= \sum _{n\ge m} a_n z^n. $$

This means, for all $\tau$ with $\Im (\tau )$ sufficiently large we have

3 $$\begin{aligned} f(\tau )=\tilde{f}(q_w). \end{aligned}$$

*Local expansions* $(g,h,\Gamma ; Y)$. Given $g(\tau )\in M_{2}(\Gamma ; \chi )$ such that ${{\,\textrm{ord}\,}}_\infty (g)\ge 0$, and $h(\tau )\in M_{0}(\Gamma )$ such that $\tilde{h}(z)=z+a_2 z^2+a_3 z^3+O(z^4)$. Then there exists a local expansion such that for all $\tau \in \mathbb H$ with $\Im (\tau )$ sufficiently large,

4 $$\begin{aligned}&g(\tau )=Y(h(\tau )) \,\text { where }\, Y(z)=\sum _{n= 0}^\infty c(n) z^n \\&\text {with}\, (c(n))_{n\ge 0} \,\text { being a holonomic sequence.} \nonumber \end{aligned}$$

It is a non-trivial fact that the *c*(*n*) indeed form a *holonomic* sequence, see the discussion related to Proposition 6.2 in [21].

### Definition 2.2

A tuple $(g,h, \Gamma ; Y)$ is called a *local expansion* if the series *Y* relates $g\in M_2(\Gamma ; \chi )$ and $h\in M_0(\Gamma )$ as in (4).

*The Sato Eigenvalue Problem (Sato-EVP)*. Given $\Gamma$ as a congruence subgroup of $\textrm{SL}_2(\mathbb {Z})$, or as a subgroup of $\textrm{GL}_2^{+}(\mathbb Q)$ being an extension of a congruence subgroup; find $$N\in \mathbb Z_{\ge 2}, \gamma _N=\left( {\begin{smallmatrix} a& b\\ c& d \end{smallmatrix}} \right) \in \Gamma $$, and $\tau _N\in \mathbb H$ such that

5 $$\begin{aligned} \left( \begin{matrix} a& b\\ c& d \end{matrix} \right) \tau _N = N\, \tau _N. \end{aligned}$$

### Definition 2.3

We call $$\left( N, \gamma _N=\left( {\begin{smallmatrix} a& b\\ c& d \end{smallmatrix}} \right) , \tau _N\right) $$ as in (5) a Sato triple for $\Gamma$.

### Example 2.4

One can easily verify,

$$ (N, \gamma _N, \tau _N) = \left( 233, \left( \begin{matrix} -231& 116\\ -2& 1 \end{matrix} \right) , \frac{116+i\sqrt{58}}{233} \right) {\tiny \,\text { is a Sato triple for }}\, \Gamma :=\Gamma (2). $$

### Remark 2.5

If $\Gamma$ is a classical congruence subgroup, there are straight-forward procedures to find Sato triples computationally. For example, if $\tau _N$ solves (5), then it must be a root of the polynomial

6 $$\begin{aligned} p(\tau ) = N c \tau ^2 + N \tau d - a \tau - b; \end{aligned}$$

i.e.,

7 $$\begin{aligned} \tau = \frac{- (N d - a) \pm \sqrt{\Delta }}{2Nc} \end{aligned}$$

where $\Delta = (Nd-a)^2+4Nbc = (Nd+a)^2 -4N\det (\gamma _N)$ is the discriminant of $p(\tau )$. Since we want (non-real) solutions in $\mathbb H$, we restrict to $\Delta <0$; i.e., $v^2 < 4N\det (\gamma )$ where $v:=(dN+a) \in \mathbb Z$. For $\Gamma \le \textrm{SL}_2(\mathbb {Z})$ and fixed *N*, this gives only finitely many candidates for *v*. For each of these candidates and for each $d \in \mathbb Z$, we can find an $a:= v-dN$ and corresponding matrix entries *b* and *c* to obtain a $$\gamma _N=\left( {\begin{smallmatrix} a& b\\ c& d \end{smallmatrix}} \right) $$ with $\det (\gamma _N)=1$ such that (5).

In fact, the choice of *c* is under the condition that the expression for *b* yields an integer and $\gamma _N\in \Gamma$, the respective group under consideration; i.e., we have

8 $$\begin{aligned} \gamma _N = \left( \begin{matrix} v - d N& \frac{d(v-dN)-1}{c}\\ c& d \end{matrix} \right) . \end{aligned}$$

Hence to satisfy these conditions, we only need to try values from suitably fixed (finite) domains for *v*, *c*, and *d*. Note that two solutions $(\gamma _1,\tau _1)$ and $(\gamma _2,\tau _2)$ with $\gamma _i\tau _i=N\tau _i$, $i\in \{1,2\}$, can be considered as equivalent if $\tau _1$ and $\tau _2$ are equivalent; i.e., if there is a matrix $\gamma \in \Gamma$ with $\tau _2=\gamma \tau _1$.

### Example 2.6

Consider the following subgroup of $\textrm{GL}_2^{+}(\mathbb Q)$,

9 $$\begin{aligned} \Gamma :=\bigg \langle \Gamma (2), \left( \begin{matrix} 1& 0\\ 1& 1 \end{matrix} \right) , \left( \begin{matrix} 0& -2\\ 1& 0 \end{matrix} \right) \bigg \rangle ; \end{aligned}$$

i.e., the group generated by the elements of $\Gamma (2)$ together with $$\gamma _1:=\left( {\begin{smallmatrix} 1& 0\\ 1& 1 \end{smallmatrix}} \right) $$ and $$\gamma _2:=\left( {\begin{smallmatrix} 0& -2\\ 1& 0 \end{smallmatrix}} \right) $$. Note that $\det (\gamma _2)=2$. One can easily verify:

10 $$\begin{aligned} (N, \gamma _N, \tau _N) = \left( 29, \left( \begin{matrix} 58& -2\\ 59& -2 \end{matrix} \right) , \frac{2}{59}\frac{58+i\sqrt{58}}{58} \right) \,\text { is a Sato triple for }\, \Gamma . \end{aligned}$$

Subsequently we will use that

11 $$\begin{aligned} \left( \begin{matrix} 58& -2\\ 59& -2 \end{matrix} \right) = {\gamma _1}\, {\gamma _2}\, {\gamma _1}^{-29}\in \Gamma . \end{aligned}$$

### Remark 2.7

In case that $\Gamma$ is an extension of a classical congruence subgroup, to find Sato triples computationally one has to modify the procedure from the previous remark. If $\Gamma$ is the group of Example 2.6, then one can show that

$$\begin{aligned} \Gamma = \{4^n\gamma : n \in \mathbb Z\,\text { and }\, \gamma \in \Gamma _1 \cup \Gamma _2\}, \end{aligned}$$

where

$$\begin{aligned} \Gamma _1&= \Bigl \{\gamma \in \textrm{SL}_2(\mathbb {Z}): \gamma \equiv \left( \begin{matrix} 1& 0\\ *& 1 \end{matrix} \right) \pmod {2}\Bigr \} \, \, \text {and}\\ \Gamma _2&= \Bigl \{\gamma \in \textrm{GL}_2^{+}(\mathbb Z): \det (\gamma )=2, \gamma \equiv \left( \begin{matrix} 0& 0\\ 1& 0 \end{matrix} \right) \pmod {2}\Bigr \}. \end{aligned}$$

Thus, one basically has to run the search twice, first for $\Gamma _1$ as in Example 2.6 and then for $\Gamma _2$ where the 1 in $(8)$ which corresponds to the determinant of $\gamma _N$ is replaced by 2.

## Algorithmization of the Sato construction described by Yang

In this section, the centerpiece of our article, a detailed algorithmic specification of the Sato construction is given. All the steps are illustrated with the incremental assembly of Ramanujan’s series (1) as a concrete running example.

### General background of Sato’s construction

Despite using a slightly more general setting, the steps below follow closely the Sato construction given by Yang [31, pp. 3–4].

Nevertheless, we introduce a significant difference to Yang’s description: *we present an algorithmic version for each of the steps*. This also motivates the following input/output specification of the procecure which we call Sato construction:

*Input.* A local expansion $(g,h,\Gamma ; Y)$ with $g\in M_2(\Gamma ; \chi )$ and $h\in M_0(\Gamma )$ as in Definition 2.2; i.e., for all $\tau \in \mathbb H$ with $\Im (\tau )$ sufficiently large,

12 $$\begin{aligned} g(\tau )=Y(h(\tau )) \,\text { where }\, Y(z)=\sum _{n= 0}^\infty c(n) z^n \end{aligned}$$

for some holonomic sequence *c*(*n*). In addition, we require that we are able to compute sufficiently many coefficients of the series representations $\tilde{g}(x)$ and $\tilde{h}(x)$.

*Output.* Integers *A*, *B*, an algebraic number $C\in \mathbb Q[\zeta ]$, and an algebraic number $t\in \mathbb Q[\xi ]$ such that

13 $$\begin{aligned} \frac{1}{\pi } = C\cdot \sum _{n=0}^{\infty } (A n+B) c(n) t^n. \end{aligned}$$

In the literature one finds various notions of “Ramanujan–Sato” series. In this article, the term *Ramanujan–Sato series* is reserved for series of the type as on the right side of (13) which, as an additional requirement, can be obtained by the Sato construction.

#### Example 3.1

We will show that Ramanujan’s series (1), which can be rewritten as

14 $$\begin{aligned} \frac{1}{\pi } = \frac{2\sqrt{2}}{9801}\cdot \sum _{n=0}^\infty (26390 n+1103) \frac{ (1/4)_n(1/2)_n (3/4)_n}{(1)_n (1)_n n!} 256^n \left( \frac{1}{396^4} \right) ^n, \end{aligned}$$

is indeed a Ramanujan–Sato series. To start the algorithmic construction of  (14), we take as input $(g,h,Y;\Gamma )$ where

15 $$\begin{aligned} \Gamma&:=\Gamma (2), \end{aligned}$$

16 $$\begin{aligned} g(\tau )&:=(1+\lambda (\tau ))\theta _3(\tau )^4 \in M_2(\Gamma ), \end{aligned}$$

17 $$\begin{aligned} h(\tau )&:= \frac{ \lambda (\tau ) (1-\lambda (\tau ))^2 }{ 16 (1+\lambda (\tau ))^4 } \in M_0(\Gamma ), \end{aligned}$$

and

18 $$\begin{aligned} Y(z):= \sum _{n=0}^\infty \underbrace{\frac{ (1/4)_n(1/2)_n (3/4)_n}{(1)_n (1)_n n!} 256^n}_{=c(n)} \cdot z^n. \end{aligned}$$

The corresponding relation $g(\tau )=Y(h(\tau ))$ is a rewritten version of the Borwein relation [4, Thm. 5.7b(iv)]; for our rewriting we use the Jacobi theta function $\theta _3(\tau ):= \theta _3(0,\tau )$ and the modular lambda function $\lambda (\tau )$. For definitions and properties of these functions we refer to the open source library FunGrim [19] which we found very useful for the applications under discussion. For example, FunGrim at one place provides all the function properties needed to verify the memberships $g\in M_2(\Gamma (2))$ and $h\in M_0(\Gamma (2))$.

Particularly relevant to the algorithmic construction of Ramanujan–Sato series is the fact that given $(g,h,\Gamma )$, the holonomic coefficient sequence *c*(*n*) of *Y*(*z*) can be determined also in an algorithmic fashion; see Sect. 10.

#### Remark 3.2

The prominence of (1), resp. (14), is owing to the fact that in 1985 Bill Gosper was successful in computing more than 17 million digits of $\pi$ using (14). We present a quote from Richard Askey’s insightful and also entertaining review [1] of the book [4] by Jon and Peter Borwein: “Gosper asked if I knew how to prove (14), and I had to admit I did not. Ramanujan had not given a proof. $[\dots ]$ If you are curious about how to prove (14), an outline is given in Chapter 5 of [4].”

Applying Step 1 to Step 8 of the Sato construction to our running example, we will derive *and* prove Ramanujan’s series (14) used by Gosper.

### Step 0: start with a local expansion

The starting point of the Sato construction is an input in the form of a local expansion $(g,h,\Gamma ; Y)$ as in (12) where we added the requirement that we are able to compute sufficiently many coefficients in the series representations,

$$ \tilde{g}(x):= \sum _{n\ge 0} g_n x^n \,\textit{ and }\, \tilde{h}(x):= x+\sum _{n\ge 2} h_n x^n, $$

where

$$ x=q_w=\exp (2 \pi i \tau /w). $$

Recall that the equalities,

19 $$\begin{aligned} g(\tau )=\sum _{n\ge 0} g_n \exp (2 \pi i \tau /w)^n (=\tilde{g}(x)) \,\textit{ and }\, h(\tau )=\sum _{n\ge 0} h_n \exp (2 \pi i \tau /w)^n (=\tilde{h}(x)), \end{aligned}$$

in general hold only when $\Im (\tau )$ is sufficiently large.

#### Example 3.3

*(Ex.* 3.1*contd.)* We proceed with the input data from Example 3.1 to illustrate the steps of the construction. At this point, we set up the corresponding *x*-series. We have $w=2$; i.e.,

$$ x=q_2=\exp (\pi i \tau ). $$

With this setting one has the classical product representation for $\lambda$; e.g. [19]:

20 $$\begin{aligned} \lambda (\tau )&= \frac{\theta _2(\tau )^4}{\theta _3(\tau )^4} \in {M_0(\Gamma (2))}\\&=16 x \Bigg [ \frac{\prod _{n=1}^{\infty }(1-x^{2n})(1+x^{2n})^{2}}{\prod _{n=1}^{\infty }(1-x^{2n})(1+x^{2n-1})^{2}} \Bigg ]^4 \nonumber \\&= 16 x - 128 x^2 + 704 x^3 - 3072 x^4 + O(x^5) = \tilde{\lambda }(x). \nonumber \end{aligned}$$

This gives the required *x*-series representation for $h(\tau )=\frac{\lambda (\tau )(1-\lambda (\tau ))^2}{16(1+\lambda (\tau ))^4}$,

21 $$\begin{aligned} \tilde{h}(x)= x - 104 x^2 + 6444 x^3 - 311744 x^4 + 13018830 x^5 +O(x^{6}) \end{aligned}$$

and similarly also the one for $g(\tau )= (1+\lambda (\tau )) \theta _3(\tau )^4$,

22 $$\begin{aligned} \tilde{g}(x)= 1+24 x+24 x^2 +96 x^3 + 24 x^4 + 144 x^5 +O(x^6). \end{aligned}$$

The *x*-series representations of *g* and *h* are taken as input to compute the local expansion $(g,h,\Gamma ; Y)$.

For example, with the *x*-series (21) and (22) in hand, one determines that

23 $$\begin{aligned} c(n)=\frac{ (1/4)_n(1/2)_n (3/4)_n}{(1)_n (1)_n n!} 256^n =\frac{(4n)!}{(n!)^4} \end{aligned}$$

such that

24 $$\begin{aligned} g(\tau ) = \sum _{n=0}^\infty c(n)h(\tau )^n \,\text { for }\, \Im (\tau ) \,\text { sufficiently large}; \end{aligned}$$

i.e., *Y*(*z*) is as in (18). How this is done algorithmically is described in Sect. 10.

Now we are ready to proceed with the actual steps of the Sato construction.

### Step 1: implement the Sato function *G*

Define

25 $$\begin{aligned} G(\tau ) := \frac{w}{2 \pi i}\, \frac{g'(\tau )}{g(\tau )} = x\, \frac{ \tilde{g}'(x)}{\tilde{g}(x)} = \tilde{G}(x). \end{aligned}$$

The function *G* is not a modular but a quasi-modular form:

#### Lemma 3.4

For all $$\gamma =\left( {\begin{smallmatrix} a& b\\ c& d \end{smallmatrix}} \right) \in \Gamma $$,

26 $$\begin{aligned} G(\gamma \tau ) = \frac{w c}{\pi i\cdot \det (\gamma )}\, (c \tau +d)+ \frac{1}{\det (\gamma )} (c \tau + d)^2 G(\tau ). \end{aligned}$$

#### Proof

Obviously,

27 $$\begin{aligned} \frac{d}{d\tau } g(\gamma \tau ) = g'(\gamma \tau ) \frac{d}{d\tau } (\gamma \tau ) = g'(\gamma \tau )\, \frac{\det (\gamma )}{(c \tau +d)^2}. \end{aligned}$$

Owing to $g\in M_2(\Gamma ; \chi )$, the left hand side equals

$$\begin{aligned} \frac{d}{d\tau } g(\gamma \tau )&= \frac{d}{d\tau } \frac{\chi (\gamma )}{\det (\gamma )} (c \tau + d)^2 g(\tau )\\&= \frac{\chi (\gamma )}{\det (\gamma )}\, \left( 2 c (c \tau +d) g(\tau ) + (c \tau +d)^2 g'(\tau ) \right) . \end{aligned}$$

This implies,

$$\begin{aligned} \frac{2 \pi i}{w}\, G(\gamma \tau )&= \frac{g'(\gamma \tau )}{g(\gamma \tau )} = \frac{(c \tau +d)^2}{g(\gamma \tau )} \frac{\chi (\gamma )}{\det (\gamma )^2}\, \left( 2 c (c \tau +d)g(\tau ) + (c \tau +d)^2 g'(\tau )\right) \\&=\frac{1}{\det (\gamma )} \left( 2 c (c \tau +d) + (c \tau +d)^2 \frac{g'(\tau )}{g(\tau )} \right) , \end{aligned}$$

which proves the statement. $\square$

#### Example 3.5

*(Ex.* 3.1*contd.)* The series expansion of $\tilde{g}(x)$ from (22) gives,

28 $$\begin{aligned} \tilde{G}(x) = x \frac{\frac{d}{dx}\big ( 1+24 x + 24 x^2 + 96 x^3+O(x^4) \big )}{1+24 x + 24 x^2 + 96 x^3+O(x^4)}= {24 x -528 x^2+ O(x^3).} \end{aligned}$$

### Step 2: implement the Sato function *H*

The definition of the Sato *H* function is with respect to a fixed integer $N\ge 2$.

#### Definition 3.6

Define

29 $$\begin{aligned} H(\tau ):=G(\tau )- N\, G(N \tau )= \tilde{G}(x)-N\, \tilde{G}(x^N) = \tilde{H}(x) \end{aligned}$$

#### Lemma 3.7

Let

30 $$\begin{aligned} \Gamma ':=\Big \{\left( \begin{matrix} a& b\\ c N& d \end{matrix} \right) \in \Gamma : \left( \begin{matrix} a& b N\\ c& d \end{matrix} \right) \in \Gamma \Big \}\le \Gamma . \end{aligned}$$

Then for all $$\left( {\begin{smallmatrix} a& b\\ c& d \end{smallmatrix}} \right) \in \Gamma '$$,

31 $$\begin{aligned} H\left( \left( \begin{matrix} a& b\\ c& d \end{matrix} \right) \tau \right) =\frac{1}{a d- b c} (c \tau + d)^2 H(\tau ). \end{aligned}$$

This means, $H\in M_2(\Gamma ')$ is a modular form of weight 2 with trivial character.

#### Proof

For $$\gamma =\left( {\begin{smallmatrix} a& b\\ c& d \end{smallmatrix}} \right) \in \Gamma '$$ one has

$$\begin{aligned} G(N \gamma \tau )&= G\left( \frac{a(N \tau )+ N b}{\frac{c}{N} (N \tau )+d} \right) =G\left( \left( \begin{matrix} a& b N\\ \frac{c}{N}& d \end{matrix} \right) (N\tau ) \right) \\&{\mathop {=}\limits ^{(26)}} \frac{w \frac{c}{N}}{\pi i\cdot \det (\gamma )}\, (c\tau +d)+ \frac{1}{\det (\gamma )} (c \tau + d)^2 G(N \tau ) \end{aligned}$$

Consequently, applying (26) again,

$$\begin{aligned} H(\gamma \tau )&= G(\gamma \tau )-N\, G(N \gamma \tau )\\&= \frac{w c}{ \pi i\cdot \det (\gamma )}\, (c \tau +d)+ \frac{1}{\det (\gamma )} (c \tau + d)^2 G(\tau )\\&\ \hspace{0.5cm}- N \frac{w \frac{c}{N}}{ \pi i\cdot \det (\gamma )}\, ( c \tau +d)- N \frac{1}{\det (\gamma )} ( c \tau + d)^2 G(N \tau )\\&= \frac{1}{\det (\gamma )} (c \tau + d)^2 \underbrace{\left( G(\tau )- N G(N \tau )\right) }_{=H(\tau )}. \end{aligned}$$

This proves the statment. $\square$

#### Example 3.8

*(Ex.* 3.1*contd.)* With the choice $N=29$ the series expansion of $\tilde{G}(x)$ from (28) gives,

32 $$\begin{aligned} \tilde{H}(x) = \tilde{G}(x)-29 \, \tilde{G}(x^{29}) =24 x -528 x^2+ 12384 x^3 +O(x^4). \end{aligned}$$

### Step 3: find a sato triple for $\Gamma$

Suppose one finds a Sato triple $(N, \gamma _N, \tau _N)$ for $\Gamma$, then

33 $$\begin{aligned} G(\gamma _N \tau _N) = G(N \tau _N), \end{aligned}$$

which allows for a crucial interplay between *G* and *H*.

#### Lemma 3.9

(Sato relation for $1/\pi$) Given a modular form $g\in M_2(\Gamma ; \chi )$ and a modular function $h\in M_0(\Gamma )$. Let

34 $$\begin{aligned} \left( N, \gamma _N=\left( \begin{matrix} a& b\\ c& d \end{matrix} \right) , \tau _N\right) \,\text { be a Sato triple for }\, \Gamma . \end{aligned}$$

Then for the associated Sato functions *G* and *H* defined in (25) and (3.6), where the *N* from (34) is used to define *H*, one has

35 $$\begin{aligned} \frac{1}{\pi } = \alpha \, G(\tau _N) + \beta \, H(\tau _N), \end{aligned}$$

where

36 $$\begin{aligned} \beta = - \frac{i}{w c N} \cdot \frac{\det (\gamma _N)}{c \tau _N +d} \,\,\text { and }\,\, \alpha = - \frac{i}{w c}\cdot (c \tau _N +d)- \beta . \end{aligned}$$

#### Proof

We begin with a rewritten version of (26),

$$\begin{aligned} - \frac{w c}{ \pi i}\, (c \tau _N +d)&=(c \tau _N + d)^2 G(\tau _N) - \det (\gamma _N)\, G(\gamma _N \tau _N) \\&{\mathop {=}\limits ^{(33)}} (c \tau _N + d)^2 G(\tau _N) - \det (\gamma _N)\, G(N \tau _N)\\&{\mathop {=}\limits ^{(29)}} \left( (c \tau _N + d)^2 -\frac{\det (\gamma _N)}{N} \right) G(\tau _N) + \frac{\det (\gamma _N)}{N} H(\tau _N). \end{aligned}$$

Rearranging terms gives (35) together with (36). $\square$

#### Example 3.10

*(Ex. *3.1*contd.)* Recall from Example 2.4 the Sato triple

37 $$\begin{aligned} (N, \gamma _N, \tau _N) = \left( 233, \left( \begin{matrix} -231& 116\\ -2& 1 \end{matrix} \right) , \frac{116+i\sqrt{58}}{233} \right) \,\text { for }\, \Gamma :=\Gamma (2). \end{aligned}$$

Using Mathematica and its built-in functions for modular $\lambda (\tau )$ and $\theta _3(z,\tau )$, we verify numerically the Sato relation (35) for $g(\tau )=(1+\lambda (\tau ))\theta _3(\tau )^4 \in M_2(\Gamma (2))$ as in (16):
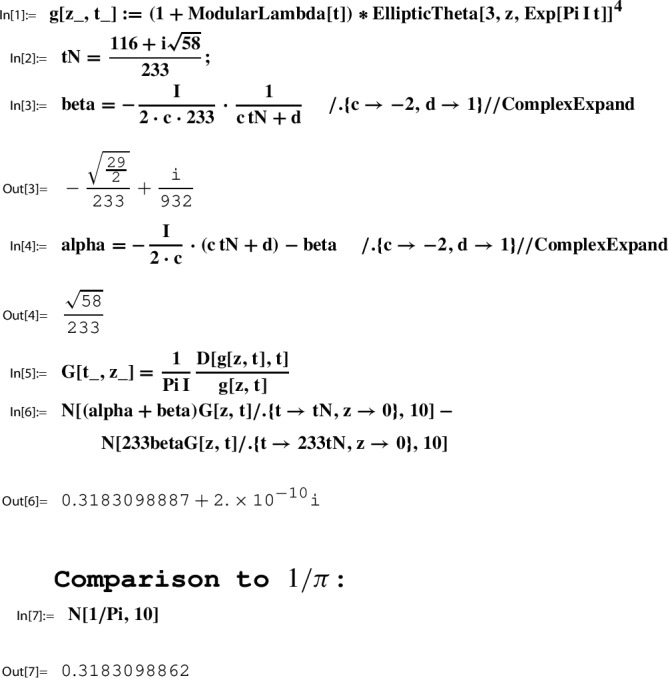


#### Example 3.11

*(Ex. *3.1*contd.)* Recall from Example 2.6 the Sato triple

38 $$\begin{aligned}&(N, \gamma _N, \tau _N) = \left( 29, \left( \begin{matrix} 58& -2\\ 59& -2 \end{matrix} \right) , \frac{2}{59}\frac{58+i\sqrt{58}}{58} \right) \end{aligned}$$

39 $$\begin{aligned}&\,\text { for }\, \Gamma :=\bigg \langle \Gamma (2), \left( \begin{matrix} 1& 0\\ 1& 1 \end{matrix} \right) , \left( \begin{matrix} 0& -2\\ 1& 0 \end{matrix} \right) \bigg \rangle =\langle \Gamma (2), \gamma _1,\gamma _2\rangle . \end{aligned}$$

In Sect. 12 we show that

40 $$\begin{aligned} g(\tau )=(1+\lambda (\tau ))\theta _3(\tau )^4\in M_2(\Gamma ) \,\text { for }\, \Gamma = \langle \Gamma (2), \gamma _1,\gamma _2\rangle ; \end{aligned}$$

concretely, for all $$\gamma =\left( {\begin{smallmatrix} a& b\\ c& d \end{smallmatrix}} \right) \in \langle \Gamma (2), \gamma _1,\gamma _2\rangle $$,

41 $$\begin{aligned} g(\gamma \tau ) = \chi (\gamma ) \det (\gamma )^{-1} (c \tau +d)^2 g(\tau ), \end{aligned}$$

where the character $\chi$ is determined uniquely by

$$ \chi (\gamma _1)=1, \chi (\gamma _2)=-1, \,\text { and }\, \chi (\gamma )=1 \,\text { for all }\gamma \in \Gamma (2). $$

Let us choose the function *g* as in (40). If we now verify numerically the Sato relation (35) for the Sato triple (38) by following the analogous Mathematica steps as in Example 3.10 and with the same precision of 10 digits, the Sato relation will be computed to the numerical value 0.318214511. This in comparison to the digits of $1/\pi$ is correct only for the first three places after the decimal point.

The explanation for this numerical discrepancy lies in the following facts:

$$ \text {for }\, \tau _{29}= \frac{2}{59}\frac{58+i \sqrt{58}}{58} \,\text { one has }\, \left| e^{\pi i \tau _{29}} \right| = 0.986114\dots , $$

in contrast to the previous Sato value, where

$$ \text {for }\, \tau _{233}= \frac{116+i \sqrt{58}}{233} \,\text { one has }\, \left| e^{\pi i \tau _{233}} \right| = 0.902411 \dots , $$

which is still close but not that close to 1. In any case, this difference, looking small at the first glance, when using theta series expansions is sufficient to provide numerical problems for the evaluation of $G(\tau _{29})$.

A simple and classical way to overcome this numerical problem is to use the (quasi-) modular transformation property (26) for *G*. To this end, one takes an element $\tau _{\infty }\in \mathbb H$ which is closer to infinity (i.e., with larger imaginary part than $\tau _{29}$) which maps via an element of $\Gamma$ to $\tau _{29}$. It is convient to make such a choice as follows,

42 $$\begin{aligned} \tau _{29} = \underbrace{\left( \begin{matrix} 0& -2\\ 1& -58 \end{matrix} \right) }_{= \gamma _1^{29} \gamma _2\in \Gamma } \tau _{\infty } \,\text { where }\,\tau _{\infty } := i \sqrt{58}. \end{aligned}$$

Note that $\left| e^{\pi i \tau _{\infty }} \right| = 4.06649\dots \times 10^{-11}$. By (26),

43 $$\begin{aligned} G(\tau _{29})= G\left( \left( \begin{matrix} 0& -2\\ 1& -58 \end{matrix} \right) \tau _{\infty }\right) = \frac{\tau _{\infty }-58}{\pi i} + \frac{(\tau _{\infty }-58)^2}{2} G(\tau _{\infty }). \end{aligned}$$

Moreover, by (38),

44 $$\begin{aligned} G(29\, \tau _{29})= G\left( \left( \begin{matrix} 58& -2\\ 59& -2 \end{matrix} \right) \tau _{29}\right) = 59 \frac{59 \tau _{29}-2}{\pi i} + \frac{(59\tau _{29}-2)^2}{2} G(\tau _{29}). \end{aligned}$$

This puts us into the position to obtain numerical evaluations with high precision.
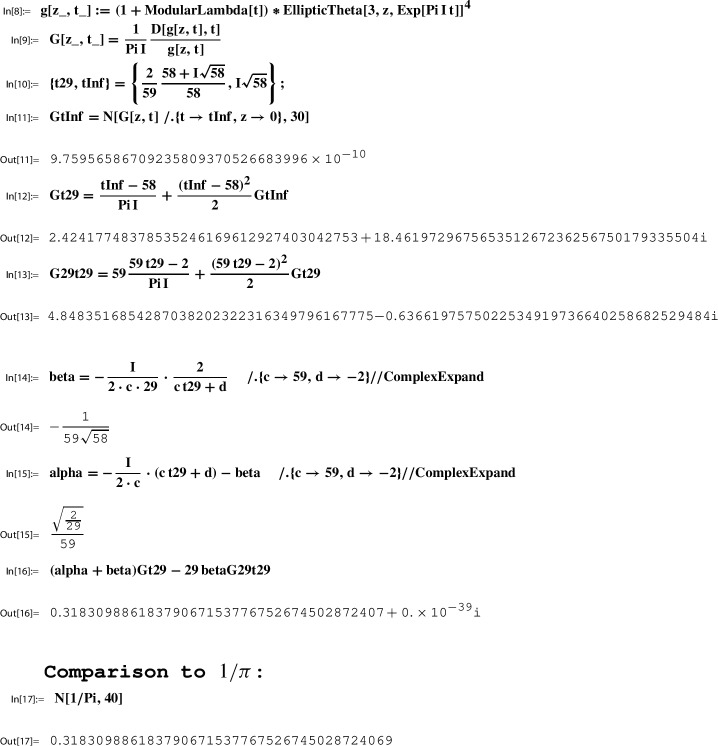


### Step 4: compute the Sato constants $\alpha$ and $\beta$

Given a Sato triple for $\Gamma$ as in (34),

$$ \left( N, \gamma _N=\left( \begin{matrix} a& b\\ c& d \end{matrix} \right) , \tau _N\right) , $$

by Lemma 3.9 the Sato constants are determined as in (36),

$$ \beta = - \frac{i}{w c N} \cdot \frac{\det (\gamma _N)}{c \tau _N +d} \,\,\text { and }\,\, \alpha = - \frac{i}{w c}\cdot (c \tau _N +d)- \beta . $$

In Example 3.10 we considered the Sato triple for $\Gamma =\Gamma (2)$, and computed in Out[3] and Out[4] the corresponding values of $\beta$ and $\alpha$, respectively.

#### Example 3.12

*(Ex. *3.1*contd.)* Example 3.11 is relevant for our running example. There we considered the Sato triple

45 $$\begin{aligned}&(N, \gamma _N, \tau _N) = \left( 29, \left( \begin{matrix} 58& -2\\ 59& -2 \end{matrix} \right) , \frac{2}{59}\frac{58+i\sqrt{58}}{58} \right) \end{aligned}$$

46 $$\begin{aligned}&\,\text { for }\, \Gamma :=\bigg \langle \Gamma (2), \left( \begin{matrix} 1& 0\\ 1& 1 \end{matrix} \right) , \left( \begin{matrix} 0& -2\\ 1& 0 \end{matrix} \right) \bigg \rangle =\langle \Gamma (2), \gamma _1,\gamma _2\rangle , \end{aligned}$$

and computed in Out[14] and Out[15],

47 $$\begin{aligned} \beta = -\frac{1}{59 \sqrt{58}} \,\text { and }\, \alpha = \frac{\sqrt{\frac{2}{29}}}{59}. \end{aligned}$$

The remaining steps of the construction deal with the conversion of the Sato relation (35) from Lemma 3.9 into a series representation for $1/\pi$, more precisely, with the conversion of $G(\tau _N)$ and $H(\tau _N)$ into series representations. Now local expansions $(g,h, \Gamma ; Y)$ come into play. Being of crucial importance, we recall the fundamental relation (4) between $g\in M_2(\Gamma ; \chi )$ and $h\in M_0(\Gamma )$:

48 $$\begin{aligned}&g(\tau )=Y(h(\tau )) \,\text { where }\, Y(z)=\sum _{n= 0}^\infty c(n) z^n \\&\text {and where }\, (c(n))_{n\ge 0} \,\text { is a holonomic sequence.} \nonumber \end{aligned}$$

In view of (48) a natural idea to represent $G(\tau _N)$ and $H(\tau _N)$ is to use the series $Y(h(\tau _N))$. So the value $h(\tau _N)$ will play an important role.

### Step 5: Compute a closed form representation of $h(\tau _N)$

To make things concrete, we continue our runnning example.

#### Example 3.13

*(Ex. *3.1*contd.)* Throughout our running example we fix *g* and *h* as in (16) and (17); i.e.,

49 $$\begin{aligned} g(\tau )&:=(1+\lambda (\tau ))\theta _3(\tau )^4 \in M_2(\Gamma (2)), \end{aligned}$$

50 $$\begin{aligned} h(\tau )&:= \frac{ \lambda (1-\lambda (\tau ))^2 }{ 16 (1+\lambda (\tau ))^4 } \in M_0(\Gamma (2)), \end{aligned}$$

In previous examples we already worked with the extended group,

51 $$\begin{aligned} \Gamma :=\bigg \langle \Gamma (2), \left( \begin{matrix} 1& 0\\ 1& 1 \end{matrix} \right) , \left( \begin{matrix} 0& -2\\ 1& 0 \end{matrix} \right) \bigg \rangle =\langle \Gamma (2), \gamma _1,\gamma _2\rangle . \end{aligned}$$

As stated explicitly in the following lemma, the modularity of *g* and *h* as in (49) and (50) extends to this bigger group, a fact which is proven in Sect. 12.

#### Lemma 3.14

We have,

52 $$\begin{aligned} g\in M_2(\Gamma ; \chi ) \,\text { and }\, h\in M_0(\Gamma ) \,\text { where }\, \Gamma = \langle \Gamma (2), \gamma _1,\gamma _2\rangle , \end{aligned}$$

and where the character $\chi$ is determined uniquely by

$$ \chi (\gamma _1)=1, \chi (\gamma _2)=-1, \,\text { and }\, \chi (\gamma )=1 \,\text { for all }\gamma \in \Gamma (2). $$

Consider the Sato triple (45) for $\Gamma$ as in (46). By Lemma 3.14 and (42) we have for $$\gamma _1^{29} \gamma _2 = \left( {\begin{smallmatrix} 0& -2\\ 1& -58 \end{smallmatrix}} \right) \in \Gamma $$,

53 $$\begin{aligned} h(\tau _{29}) = h( \gamma _1^{29} \gamma _2\ i \sqrt{58}) = h(i \sqrt{58}). \end{aligned}$$

Next we determine this value numerically using ModularLambda, the built-in function Mathematica provides for $\lambda$:
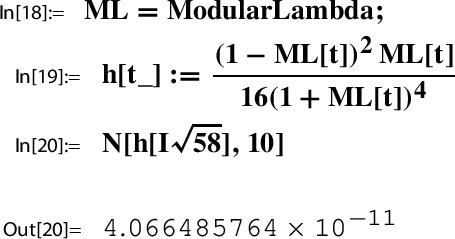
 The output indicates that $h(i \sqrt{58})$ is a real number. In view of $10^{-11}$ we look at the reciprocal value: 
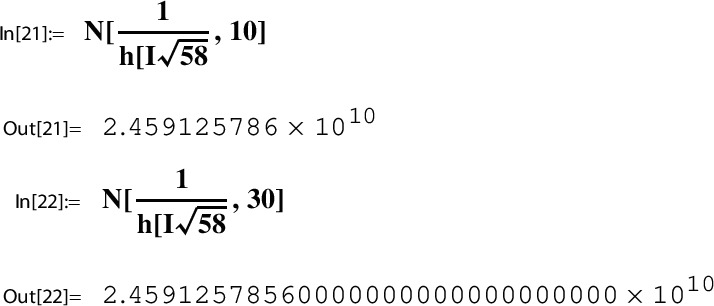
 The numerical stability displayed by the last output line suggests that $h(i \sqrt{58})$ is a rational number.

#### Lemma 3.15

For *h* as in (50) and $\tau _{29}$ as in (45),

54 $$\begin{aligned} h(\tau _{29})=h(i \sqrt{58})= \frac{1}{2459125786} = \frac{1}{396^4}. \end{aligned}$$

The proof of this lemma together with remarks on related computational aspects is given in Sect. 8. Our running example will be continued in the next section, Step 6.

In general, closed form representations of $h(\tau _N)$ depend on the functions defining *h*, and on available $\tau _N$ from Sato triples from which suitable candidates are chosen. To this end, lists as presented at [29] or [30] are helpful.

If no further information on such $\tau _N$ is available, one, in a first step, can try to derive a plausible guess for $h(\tau _N)$ numerically. Then to prove a conjectured closed form representation of $h(\tau _N)$, one either uses classical methods or algorithmic tools. Concerning the latter, further remarks are given in Sect. 8; see, in particular, Sect. 8.1 for an algorithmic derivation and proof of (54) done with the help of the MultiSamba algorithm.

### Step 6: compute a series representation of $H(\tau _N)$

The verification of the following fact is straight-forward.

#### Lemma 3.16

For $g\in M_2(\Gamma ; \chi )$ and $H \in M_2(\Gamma ')$ as in (29), with $\Gamma '$ as in (30),

55 $$\begin{aligned} \frac{H(\tau )}{g(\tau )} \in M_0(\Gamma _\chi ^{\prime }) \end{aligned}$$

where

$$ \Gamma _\chi ^{\prime }:=\Big \{\left( \begin{matrix} a& b\\ c& d \end{matrix} \right) \in \Gamma ': \chi (a,b,c,d)=1 \Big \}. $$

As a modular function with respect to $\Gamma _\chi ^{\prime }$, the function *H*/*g* satisfies an algebraic relation with $h\in M_0(\Gamma )$; notice that $h\in M_0(\Gamma _\chi ^{\prime })$ owing to $\Gamma _\chi ^{\prime }\le \Gamma '\le \Gamma$. Consequently, *H*/*g* can be considered as an algebraic function in *h*, which justifies the notation

56 $$\begin{aligned} p_1(h(\tau )):= \frac{H(\tau )}{g(\tau )}, \end{aligned}$$

where $p_1$ denotes the corresponding algebraic function.

One essential ingredient to Step 6 is to invoke the local expansion as in (4); i.e., for $g(\tau )\in M_{2}(\Gamma ; \chi )$ and $h(\tau )\in M_{0}(\Gamma )$,

57 $$\begin{aligned}&g(t)=Y(h(t)) \,\text { where }\, Y(z)=\sum _{n= 0}^\infty c(n) z^n, \end{aligned}$$

which in general holds only for such $t\in \mathbb H$ where $\Im (t)$ is sufficiently large. In order to invoke (57) for concrete evaluation points *t*, one needs to determine a concrete neighborhood of infinity; i.e., a lower bound $L>0$ such that (57) holds for all $t\in \mathbb H$ with $\Im (t)>L$. As a concrete example, in Sect. 11 we derive and prove the bound $L=1.87$ for the local expansion (24) of our running example.

It can happen that the $\tau _N$ from a Sato triple does not lie in a neighborhood $U_L$ of infinity induced by such a lower bound *L*. In such cases one can still proceed with the Sato construction if a point *t* equivalent to $\tau _N$ exists in $U_L$. In other words, if there exists a transformation $T\in \Gamma$ which allows to increase the imaginary part of $\tau _N$ such that

58 $$\begin{aligned} \text {for }\, t:= T \tau _N\in \mathbb H\,\text { the local expansion (57) holds}. \end{aligned}$$

Suppose we have such a *t*, then setting $$S:=T^{-1}=\left( {\begin{smallmatrix} s_1& s_2\\ s_3& s_4 \end{smallmatrix}} \right) $$,

$$\begin{aligned} H(\tau _N)&=\frac{H(\tau _N)}{g(\tau _N)}\cdot g(\tau _N) = p_1(h(\tau _N))\cdot g(S t)\\&=p_1(h(\tau _N))\cdot \chi (S) \det (S)^{-1} (s_3 t + s_4)^2 g(t) \\&{\mathop {=}\limits ^{(58)}} p_1(h(\tau _N))\cdot \chi (S) \det (S)^{-1} (s_3 t + s_4)^2 \sum _{n= 0}^\infty c(n) h(t)^n\\&= \frac{\chi (S)(s_3 T \tau _N + s_4)^2}{ \det (S)} \cdot p_1(h(\tau _N)) \sum _{n= 0}^\infty c(n) h(\tau _N)^n, \end{aligned}$$

where the last equality is by $h(t)=h(T \tau _N) =h(\tau _N)$.

The occurence of $p_1$ reveals also the second essential ingredient to Step 6:

$$ \text {determine a closed form for}\, p_1(h(\tau _N)). $$

Using our toolbox, also this task, in principle, can be done algorithmically. A description of the corresponding procedure is given in Sect. 9.1.

*Summary of Step 6:* First, one has to accomplish the following task:

59 $$\begin{aligned}&\text {Determine a bound }L>0\text { such that for all }\, t\in \mathbb H\,\text { with }\, \Im (t)>L,\nonumber \\&g(t)=Y(h(t)) \,\text { where }\, Y(z)=\sum _{n= 0}^\infty c(n) z^n. \end{aligned}$$

Next, determine $$S=T^{-1}=\left( {\begin{smallmatrix} s_1& s_2\\ s_3& s_4 \end{smallmatrix}} \right) $$ such that $\Im (T \tau _N)>L$, which implies that

60 $$\begin{aligned} H(\tau _N) = \frac{\chi (S)(s_3 T \tau _N + s_4)^2}{ \det (S)} \cdot p_1(h(\tau _N)) \sum _{n= 0}^\infty c(n) h(\tau _N)^n, \end{aligned}$$

with $p_1(h(\tau _N))$ as in (56). Finally, determine a closed form for $p_1(h(\tau _N))$.

#### Example 3.17

*(Ex. *3.1*contd.)* We continue our running example; i.e., with *g* and *h* and the Sato triple $(29, \gamma _{29}, \tau _{29})$ as in (49), (50), and (45), respectively. Recall that for all $$\gamma =\left( {\begin{smallmatrix} a& b\\ c& d \end{smallmatrix}} \right) \in \Gamma :=\langle \Gamma (2), \gamma _1,\gamma _2\rangle $$,

61 $$\begin{aligned} g(\gamma \tau ) = \chi (\gamma ) \det (\gamma )^{-1} (c \tau +d)^2 g(\tau ), \end{aligned}$$

where the character $\chi$ is determined uniquely by

62 $$\begin{aligned} \chi (\gamma _1)=1, \chi (\gamma _2)=-1, \,\text { and }\, \chi (\gamma )=1 \,\text { for all }\gamma \in \Gamma (2). \end{aligned}$$

In addition, recall (42),

63 $$\begin{aligned} \tau _{29} = \underbrace{\left( \begin{matrix} 0& -2\\ 1& -58 \end{matrix} \right) }_{= \gamma _1^{29} \gamma _2\in \Gamma } \tau _{\infty } \,\text { where }\,\tau _{\infty } := i \sqrt{58}, \end{aligned}$$

and (54),

$$ h(\tau _{29})= h(i \sqrt{58})=\frac{1}{396^4}. $$

In Sect. 11 we proved $L=1.87$ as a bound for the local expansion,

64 $$\begin{aligned} g(t)=\sum _{n=0}^\infty \frac{ (1/4)_n(1/2)_n (3/4)_n}{(1)_n (1)_n n!} 256^n h(t)^n,\, \, \Im (t)>1.87. \end{aligned}$$

Observing that

$$ \Im (\tau _{29})=0.0044\dots \,\text { and }\, \Im (\tau _{\infty })=7.61577\dots > 1.87, $$

and in view of $$\left( {\begin{smallmatrix} 0& -2\\ 1& -58 \end{smallmatrix}} \right) ^{-1} \tau _{29}= \tau _{\infty }$$ we apply (60) with

65 $$\begin{aligned} T:=\left( \begin{matrix} 0& -2\\ 1& -58 \end{matrix} \right) ^{-1} = \left( \begin{matrix} -29& 1\\ -\frac{1}{2}& 0 \end{matrix} \right) \,\text { and }\, S:=T^{-1} = \left( \begin{matrix} 0& -2\\ 1& -58 \end{matrix} \right) =\gamma _1^{29} \gamma _2. \end{aligned}$$

Substituting into (60) gives,

66 $$\begin{aligned} H(\tau _{29}) = p_1\left( \frac{1}{396^4}\right) \cdot (-1) \frac{1}{2} (i\sqrt{58} -58)^2 \sum _{n= 0}^\infty c(n) \left( \frac{1}{396^4}\right) ^n. \end{aligned}$$

Finally, we take the quotient with numerator

$$ \texttt {Gt29 - 29}\,\texttt { G29t29}, $$

the value representing $H(\tau _{29})$ in In[16], and with denominator being a truncated version of the series to the right of (66) to conjecture a closed form for $p_1(h(\tau _{29}))=p_1(h(1/396^4))$. Recall that $\texttt {tInf}=i \sqrt{58}$ has been defined in In[10]:
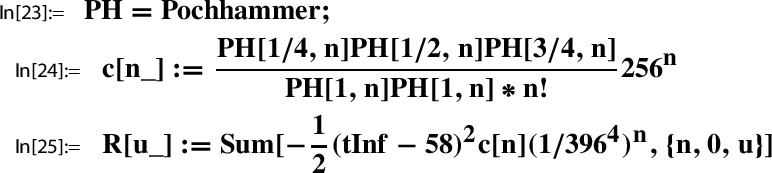
 Using Gt29 and G29t29 from the Mathematica session in Example 3.13, we obtain a numerical value for $p_1\left( \frac{1}{396^4}\right)$: 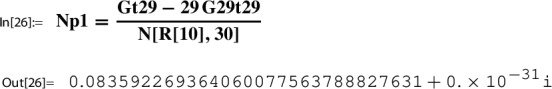 Finally, we use Mathematica to guess an algebraic representation of $p_1\left( \frac{1}{396^4}\right)$: 
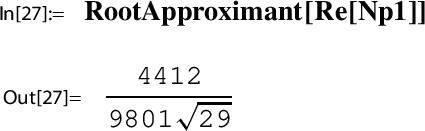


As described in Sect. 9.1, using the algorithm MultiSamba one can not only find but also prove this evaluation which we state as a lemma.

#### Lemma 3.18

Let *g* and *h* and the Sato triple $(29, \gamma _{29}, \tau _{29})$ be as in Example 3.17. Define the algebraic function $p_1$ as above by

$$ p_1(h(\tau )):= \frac{H(\tau )}{g(\tau )}. $$

Then

67 $$\begin{aligned} p_1(h(\tau _{29})) = p_1(h(\tau _{\infty })) = p_1\left( \frac{1}{396^4}\right) = \frac{4412}{9801 \sqrt{29}}. \end{aligned}$$

### Step 7: compute a series representation of $G(\tau _N)$

Step 7 works very similarly to Step 6.

#### Lemma 3.19

Let $(g,h,\Gamma ; Y)$ be a local expansion as in Definition 2.2, and let *G* be the associated Sato function as in (25). Then for all $\tau \in \mathbb H$ such that $\Im (\tau )$ sufficiently large,

68 $$\begin{aligned} G(\tau )= \frac{w}{2 \pi i} \frac{{h'}(\tau )}{g(\tau )} \cdot Y'(h(\tau )). \end{aligned}$$

#### Proof

The chain rule used on (4),

$$ {g'}(\tau ) = \frac{dY(h(\tau ))}{d\tau } = Y'(h(\tau ))\cdot {h'}(\tau ), $$

gives

$$ G(\tau ) =\frac{w}{2 \pi i}\, \frac{{g'}(\tau )}{g(\tau )} = \frac{w}{2 \pi i} \frac{{h'}(\tau )}{g(\tau )} \cdot Y'(h(\tau )). $$

$$\square $$

Also the next lemma is straight-forward to prove.

#### Lemma 3.20

Let

$$ \Gamma _\chi :=\Big \{\left( \begin{matrix} a& b\\ c& d \end{matrix} \right) \in \Gamma : \chi ( a, b, c, d) =1 \Big \}, $$

then

$$ \frac{{h'}(\tau )}{g(\tau )} \in M_0(\Gamma _\chi ). $$

#### Proof

For $$\gamma =\left( {\begin{smallmatrix} a& b\\ c& d \end{smallmatrix}} \right) \in \Gamma _\chi $$,

$$\begin{aligned} \frac{{h'}(\gamma \tau )}{g( \gamma \tau )}&= \frac{(c \tau +d)^2}{\det (\gamma )} \frac{d}{d\tau } h(\gamma \tau ) \cdot \frac{1}{g(\gamma \tau )} = \frac{(c \tau +d)^2}{\det (\gamma )} {h'}(\tau ) \cdot \frac{1}{g(\gamma \tau )}\\&= \frac{(c \tau +d)^2}{\det (\gamma )} h'(\tau ) \cdot \frac{1}{\chi (\gamma )} \frac{\det (\gamma )}{(c \tau +d)^2} \frac{1}{g(\tau )} = \frac{{h'}(\tau )}{g(\tau )}. \end{aligned}$$

$$\square $$

As a modular function with respect to $\Gamma _{\chi }$, the function $h'/g$ satisfies an algebraic relation with $h\in M_0(\Gamma )$; notice that $h\in M_0(\Gamma _\chi )$ owing to $\Gamma _\chi \le \Gamma$. Consequently, $h'/g$ can be considered as an algebraic function in *h*, which justifies the notation

69 $$\begin{aligned} p_2(h(\tau )):= \frac{w}{2 \pi i} \,\frac{h'(\tau )}{g(\tau )}, \end{aligned}$$

where $p_2$ denotes the corresponding algebraic function.

As in Step 6, for $\tau _N$ taken from a Sato triple $(N, \gamma _N, \tau _N)$ for $\Gamma$, let $T\in \Gamma$ be a transformation such that

$$\begin{aligned} \text {for }\, t:= T \tau _N\in \mathbb H\,\text { the local expansion (57), respectively (4), holds}. \end{aligned}$$

For such a *t* the relation (68) holds; i.e.,

$$ G(t)= p_2(h(t)) \cdot Y'(h(t)). $$

Then, setting $$S:=T^{-1}=\left( {\begin{smallmatrix} s_1& s_2\\ s_3& s_4 \end{smallmatrix}} \right) $$, by (26),

$$\begin{aligned} G(\tau _N)&= G(S t) = \frac{w s_3}{\pi i\cdot \det (S)}\, (s_3 t +s_4)+ \frac{1}{\det (S)} (s_3 t + s_4)^2 G(t)\\&= \frac{w s_3(s_3 T \tau _N +s_4)}{\pi i\cdot \det (S)}\, + \frac{(s_3 T \tau _N + s_4)^2}{\det (S)}\, p_2(h(\tau _N)) \cdot Y'(h(\tau _N)). \end{aligned}$$

The occurence of $p_2$ gives rise to a task similar to Step 6:

$$ \text {determine a closed form for}\, p_2(h(\tau _N)). $$

Using our toolbox, also this task, in principle, can be done algorithmically. A description of the corresponding procedure is given in Sect. 9.2.

*Summary of Step 7:* As in Step 6, one has to accomplish the following task first:

70 $$\begin{aligned}&\text {Determine a bound }L>0\text { such that for all }\, t\in \mathbb H\,\text { with }\, \Im (t)>L, \nonumber \\&g(t)=Y(h(t)) \,\text { where }\, Y(z)=\sum _{n= 0}^\infty c(n) z^n. \end{aligned}$$

Next, determine $$S=T^{-1}=\left( {\begin{smallmatrix} s_1& s_2\\ s_3& s_4 \end{smallmatrix}} \right) $$ such that $\Im (T \tau _N)>L$, which implies that

71 $$\begin{aligned} G(\tau _N)&= \frac{w s_3(s_3 T \tau _N +s_4)}{\pi i\cdot \det (S)} \\&\hspace{0.5cm} + \frac{(s_3 T \tau _N + s_4)^2}{\det (S)}\cdot \frac{p_2(h(\tau _N))}{h(\tau _N)} \sum _{n= 0}^\infty n\, c(n) h(\tau _N)^n, \nonumber \end{aligned}$$

with $p_2(h(\tau _N))$ as in (69). Finally, determine a closed form for $p_2(h(\tau _N))$.

#### Example 3.21

*(Ex. *3.1*contd.)* We continue our running example; i.e., with *g* and *h*, the Sato triple $(29, \gamma _{29}, \tau _{29})$, and $$S:=T^{-1}=\left( {\begin{smallmatrix} 0& -2\\ 1& -58 \end{smallmatrix}} \right) $$ as in Example 3.17. Recall from there that for $t:=T \tau _{29}=\tau _{\infty }=i\sqrt{58}$ one has $\Im (t)>L=1.87$ and thus

$$ g(t)=Y(h(t))=\sum _{n=0}^\infty \frac{ (1/4)_n(1/2)_n (3/4)_n}{(1)_n (1)_n n!} 256^n h(t)^n. $$

Substituting into (71) gives,

72 $$\begin{aligned} G(\tau _{29})= \frac{i\sqrt{58} -58}{\pi i} + \frac{(i\sqrt{58} - 58)^2}{2}\cdot \frac{p_2(1/396^4)}{1/396^4} \sum _{n= 0}^\infty n\, c(n) \left( \frac{1}{396^4}\right) ^n. \end{aligned}$$

As in Example 3.17 we use this relation to conjecture a closed form for

$$ p_2(h(\tau _{29}))= p_2\left( h\left( \frac{1}{396^4}\right) \right) . $$

To this end, we define a numerical approximation Gt29 to $G(\tau _{29})$ as In[11], and a truncated version of the series to the right of (72). Recall that c[n] has been defined in In[24]: 
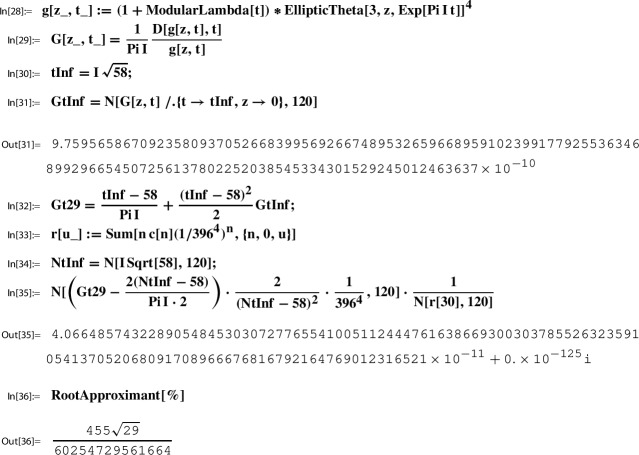


As described in Sect. 9.2, using the algorithm “MultiSamba” one can not only find but also prove this evaluation which we state as a lemma.

#### Lemma 3.22

Let *g* and *h* and the Sato triple $(29, \gamma _{29}, \tau _{29})$ be as in Example 3.17. Define the algebraic function $p_2$ as above by

$$ p_2(h(\tau )):= \frac{w}{2 \pi i} \, \frac{h'(\tau )}{g(\tau )}. $$

Then

73 $$\begin{aligned} p_2(h(\tau _{29})) = p_2(h(\tau _{\infty })) = p_2\left( \frac{1}{396^4}\right) = \frac{455 \sqrt{29}}{60254729561664}. \end{aligned}$$

### Step 8: setting up the $1/\pi$ series.

After Steps 1 to 7 we are now ready for setting up the resulting series for 1 over $\pi$. For convenience we recall the setting for our algorithmic construction.

Given $h\in M_0(\Gamma )$ and $g\in M_2(\Gamma ; \chi )$ such that for all $$\gamma =\left( {\begin{smallmatrix} A& B\\ C& D \end{smallmatrix}} \right) \in \Gamma $$,

$$ g(\gamma \tau ) = \chi (\gamma ) \det (\gamma )^{-1} (C \tau +D)^2\,g(\tau ), $$

the starting point of the Sato construction is input in the form of a local expansion $(g,h,\Gamma ; Y)$ as in (12). An additional algorithmic requirement is that we are able to compute sufficiently many coefficients of the series representations,

$$ \tilde{g}(x):= \sum _{n\ge 0} g_n x^n \,\textit{ and }\, \tilde{h}(x):= x+\sum _{n\ge 2} h_n x^n, $$

where

$$ x=q_w=\exp (2 \pi i \tau /w). $$

Recall that

$$ g(\tau )=\sum _{n\ge 0} g_n \exp (2 \pi i \tau /w)^n =\tilde{g}(x) \,\textit{ and }\, h(\tau )=\sum _{n\ge 0} h_n \exp (2 \pi i \tau /w)^n =\tilde{h}(x) $$

in general hold only when $\Im (\tau )$ is sufficiently large.

Moreover, suppose that $$(N, \gamma _N=\left( {\begin{smallmatrix} a& b\\ c& d \end{smallmatrix}} \right) , \tau _N)$$ is a Sato triple for $\Gamma$; i.e., its entries satisfy the Sato EVP (5). In addition, let $$S=T^{-1}=\left( {\begin{smallmatrix} s_1& s_2\\ s_3& s_4 \end{smallmatrix}} \right) $$ be such that for $t:=T \tau _N$ the local expansion

$$ g(t)=Y(h(t)) \,\text { with }\, Y(z)=\sum _{n= 0}^\infty c(n) z^n $$

holds. Let

$$ \beta = - \frac{i}{w c N} \cdot \frac{\det (\gamma _N)}{c \tau _N +d} \,\,\text { and }\,\, \alpha = - \frac{i}{w c}\cdot (c \tau _N +d)- \beta . $$

be as in (36), and $p_1$ and $p_2$ as in (56) and (69), respectively, then the desired series representation for 1 over $\pi$ is

74 $$\begin{aligned} \frac{1}{\pi }&= \alpha \Big ( \frac{w s_3(s_3 T \tau _N +s_4)}{\pi i\cdot \det (S)} + \frac{(s_3 T \tau _N + s_4)^2}{\det (S)}\cdot \frac{p_2(h(\tau _N))}{h(\tau _N)} \sum _{n= 0}^\infty n\, c(n) h(\tau _N)^n \Big ) \nonumber \\&+ \beta \Big ( \frac{\chi (S)(s_3 T \tau _N + s_4)^2}{ \det (S)} \cdot p_1(h(\tau _N)) \sum _{n= 0}^\infty c(n) h(\tau _N)^n \Big ). \end{aligned}$$

This relation is simply obtained from the Sato relation (35) by filling in (71) for $G(\tau _N)$ and (60) for $H(\tau _N)$.

#### Example 3.23

*(Ex. *3.1*contd.)* Now we are ready to complete our running example where

$$ x=q_2=\exp (\pi i \tau ). $$

Given the input data as in Example 3.21, we need to substitute the following items into (74):

$$\begin{aligned}&\beta = -\frac{1}{59 \sqrt{58}} \,\text { and }\, \alpha = \frac{\sqrt{\frac{2}{29}}}{59} \hspace{0.6cm}\text {(by (47))},\\&S =T^{-1} = \left( \begin{matrix} s_1& s_2\\ s_3& s_4 \end{matrix} \right) =\left( \begin{matrix} 0& -2\\ 1& -58 \end{matrix} \right) =\gamma _1^{29} \gamma _2 \hspace{0.6cm}\text { (by (65))},\\&\chi (\gamma _1)=1, \chi (\gamma _2)=-1, \,\text { and }\, \chi (\gamma )=1 \,\text { for all }\gamma \in \Gamma (2) \hspace{0.6cm}\text { (by (62))},\\&T \tau _{29} = \tau _{\infty }= i \sqrt{58} \hspace{0.6cm}\text { (by (63))},\\&h(\tau _{29})= h(i \sqrt{58})=\frac{1}{396^4} \hspace{0.6cm}\text { (by (54)) },\\&p_1(h(\tau _{29})) = \frac{4412}{9801 \sqrt{29}} \hspace{0.6cm}\text { (by (67)) },\\&p_2(h(\tau _{29})) = \frac{455 \sqrt{29}}{60254729561664} \hspace{0.6cm}\text { (by (73)) }. \end{aligned}$$

This gives

75 $$\begin{aligned} \frac{1}{\pi }&= \alpha \Bigg ( \frac{2 (i\sqrt{58} -58)}{\pi i\cdot 2} + \frac{(i\sqrt{58} -58)^2}{2}\cdot \frac{4\cdot 455 \sqrt{29}}{9801} \sum _{n= 0}^\infty n\, c(n) \left( \frac{1}{396} \right) ^{4n} \Bigg ) \nonumber \\&+ \beta \Bigg ( \frac{(-1)(i\sqrt{58} -58)^2}{ 2} \cdot \frac{4412}{9801 \sqrt{29}} \sum _{n= 0}^\infty c(n) \left( \frac{1}{396}\right) ^{4n} \Bigg ). \end{aligned}$$

Next we observe that for

$$ 29\cdot 59\cdot \Big ( \frac{1}{\pi }- \alpha \frac{ i\sqrt{58} -58}{\pi i} \Big ) \Big ( \frac{(i\sqrt{58} -58)^2}{2} \Big )^{-1}= \frac{1}{\pi }. $$

This turns (75) into

$$\begin{aligned} \frac{1}{\pi } = 29\cdot \frac{4\cdot 455 \sqrt{2}}{9801} \sum _{n= 0}^\infty n\, c(n) \left( \frac{1}{396} \right) ^{4n} + \frac{4\cdot 1103}{9801 \sqrt{2}} \sum _{n= 0}^\infty c(n) \left( \frac{1}{396}\right) ^{4n}, \end{aligned}$$

which, by recalling *c*(*n*) from (23), is Ramanujan’s series (14).

This completes the description of the steps of the Sato construction. Fundamental ingredients of this construction are solutions to the Sato Eigenvalue Problem (Sato-EVP) forming Sato triples; recall Definition  2.3. Further aspects related to these notions are discussed in Sects. 4, 5, and 6.

## Infinite families of Sato triples for Ramanujan-Gosper

The starting point, Step 0, of the Sato construction of Ramanujan’s series (1) was made by the pair

76 $$\begin{aligned} g(\tau ):=(1+\lambda (\tau ))\theta _3(\tau )^4 \in M_2(\Gamma ; \chi ) \,\text { and }\, h(\tau ) := \frac{ \lambda (\tau ) (1-\lambda (\tau ))^2 }{ 16 (1+\lambda (\tau ))^4 } \in M_0(\Gamma ), \end{aligned}$$

where

77 $$\begin{aligned} \Gamma :=\bigg \langle \Gamma (2), \left( \begin{matrix} 1& 0\\ 1& 1 \end{matrix} \right) , \left( \begin{matrix} 0& -2\\ 1& 0 \end{matrix} \right) \bigg \rangle =\langle \Gamma (2), \gamma _1,\gamma _2\rangle . \end{aligned}$$

To execute Step 0 a local expansion $(g,h,Y;\Gamma )$ was determined algorithmically, as explained in Sect. 10; i.e., the computation of a series

78 $$\begin{aligned} Y(z):= \sum _{n=0}^\infty \underbrace{\frac{ (1/4)_n(1/2)_n (3/4)_n}{(1)_n (1)_n n!} 256^n}_{=c(n)} \cdot z^n, \end{aligned}$$

with holonomic coefficient sequence (*c*(*n*)), such that for $\tau$ with $\Im (\tau )$ sufficiently large,

$$ g(\tau )=Y(h(\tau )). $$

To carry out all the remaining steps of the construction, Step 1 to Step 8, in principle, one only needs to choose a solution to the Sato-EVP; in our running example this was the Sato triple

79 $$\begin{aligned} (N, \gamma _N, \tau _N) = \left( 29, \left( \begin{matrix} 58& -2\\ 59& -2 \end{matrix} \right) , \frac{2}{59}\frac{58+i\sqrt{58}}{58} \right) \end{aligned}$$

for $\Gamma$ as in (45). From this data, all the ingredients needed to establish Ramanujan’s series (1) can be computed—primarily with the help of the algorithm MultiSamba.

A simple but far reaching observation is that a given group $\Gamma$ can give rise to parameterized families of Sato triples with infinitely many members. The next lemma presents such a family which contains (79) as the special case with $N=29$.

### Lemma 4.1

For each $N\in \mathbb Z_{\ge 2}$ one has that

80 $$\begin{aligned} (N, \gamma _N, \tau _N) = \left( N, \left( \begin{matrix} 2N& -2\\ 2N+1& -2 \end{matrix} \right) , \frac{2}{2N+1}\frac{2N+i\sqrt{2N}}{2N} \right) \end{aligned}$$

is a Sato triple for $\Gamma$ as in (77). Moreover, for $\gamma _1$ and $\gamma _2$ as in (77),

81 $$\begin{aligned} \left( \begin{matrix} 2N& -2\\ 2N+1& -2 \end{matrix} \right) = \gamma _1 \gamma _2 \gamma _1^{-N} \end{aligned}$$

and

82 $$\begin{aligned} \tau _N=\left( \begin{matrix} 0& -2\\ 1& -2N \end{matrix} \right) i \sqrt{2N} \,\text { with }\, \left( \begin{matrix} 0& -2\\ 1& -2N \end{matrix} \right) = \gamma _1^{N} \gamma _2. \end{aligned}$$

### Proof

The proof is by straight-forward verification. $\square$

Note that the instance $N=29$ of (81) and (82) corresponds to (11) and (42), respectively.

### Example 4.2

The choice $N=95$ in (80) gives the Sato triple,

83 $$\begin{aligned} (N, \gamma _N, \tau _N) = \left( 95, \left( \begin{matrix} 190& -2\\ 191& -2 \end{matrix} \right) , \frac{2}{191}\frac{190+i\sqrt{190}}{190} \right) . \end{aligned}$$

Then by (82),

$$ \tau _{95}=\left( \begin{matrix} 0& -2\\ 1& -190 \end{matrix} \right) i \sqrt{190}, $$

and for *h* as in (76) one has,

84 $$\begin{aligned} h(i \sqrt{190}) = \frac{1}{(12 \sqrt{19}\, (481 + 340 \sqrt{2}))^4} =\left( \frac{12 \sqrt{19}\, (481 - 340 \sqrt{2})}{7\cdot 9\cdot 16\cdot 19\cdot 23} \right) ^4. \end{aligned}$$

This *h* and the modular form *g* as in (76) together with the Sato triple (83) gives rise to the following approximating sum with *c*(*n*) as in (78),

85 $$\begin{aligned} \phi (m)&= \frac{\sqrt{19}}{2\cdot 3^2\cdot 7^2\cdot 19\cdot 23^2} \sum _{n=0}^m \Big ( 40 (693121 + 5457 \sqrt{2}) n \nonumber \\&\hspace{0.9cm} +(1877581 - 869892\sqrt{2}) \Big ) c(n) \left( \frac{12 \sqrt{19}\, (481 - 340 \sqrt{2})}{7\cdot 9\cdot 16\cdot 19\cdot 23} \right) ^{4n},\, \, m\ge 0. \end{aligned}$$

If *m* tends to infinity this gives a series with limit $1/\pi$; i.e.,

86 $$\begin{aligned} \frac{1}{\pi } = \lim _{m\rightarrow \infty } \phi (m). \end{aligned}$$

### Remark 4.3

Remarkably the approximating sum $\phi (m)$ adds 16 correct digits when increasing *m* incrementally in steps of 1. For example,

$$ \phi (0)=\frac{1877581-869892 \sqrt{2}}{466578 \sqrt{19}}= 0.318309886183790619\dots , $$

gives 16,

$$\begin{aligned} \phi (1)&=\frac{5 \left( 81308478120690184913-38307502735843794300 \sqrt{2}\right) }{97779701174662003392 \sqrt{19}}\\&= 0.318309886183790671537767526745027\dots , \end{aligned}$$

gives 32 correct digits of $1/\pi$, etc. The series (1) only adds 8 correct digits when adding the next summand. The celebrated Chudnovsky series  [10, Eq. (1.5)],

87 $$\begin{aligned} \frac{426880 \sqrt{10005}}{\pi } = \sum _{n=0}^\infty (545140134 n+13591409) c(n) \Big (\frac{-1}{640320}\Big )^{3n} \end{aligned}$$

adds less digits than $\phi (\infty )$, namely 14. This series has been used until today for world-record computations of digits of $\pi$; see [27].

Another aspect we want to stress is that the Sato construction with input (76), (77), (78), and the infinite family (80) of Sato triples, can be viewed as an algorithmic version of the following theorem by the Borweins:

### Theorem 4.4

([6, Thm. 5] and [4, Eq. (5.5.16)])

$$ \frac{1}{\pi } = \sum _{n=0}^\infty \frac{ (1/4)_n(1/2)_n (3/4)_n\, d_n(N)}{(n!)^3} x_N^{2n+1}, $$

where,

$$ x_N:=\frac{4 k_N (1-k_N^2)}{(1+k_N^2)^2} :=\left( \frac{g_N^{12}+g_N^{-12}}{2} \right) ^{-1}, $$

with

$$ d_n(N)= \left( \frac{\alpha (N) x_N^{-1}}{1+k_N^2} - \frac{\sqrt{N}}{4}g_N^{-12} \right) + n\sqrt{N} \left( \frac{g_N^{12}-g_N^{-12}}{2} \right) , $$

$\alpha (N)$ as in(88), and

$$ k_N=\lambda ^{*}(N):=\sqrt{\lambda (i \sqrt{N})}, \hspace{0.3cm} g_N^{12}=\frac{1-k_N^2}{2 k_N}. $$

The entities $g_N$ are called Ramanujan-Weber class invariants; they were studied independently by Ramanujan [23] and Weber [26]. The relation to one of the three Weber functions is as follows [4, Eq. (3.2.13)],

$$ g_N=2^{-1/4} f_1(\sqrt{-N}). $$

The singular value function $\alpha (r)$, $r\in \mathbb R_{>0}$, was introduced by the Borweins in [4, Eq. (5.1.1)]. The Sects. 5.1 to 5.3 of [4] are devoted to a study of its properties, including computational aspects. In [6, Eq. (7.3)] a representation in terms of an *x*-series is given,

88 $$\begin{aligned} \alpha (r)= \left( \frac{1}{\pi }-4\, \sqrt{r}\cdot \frac{\sum _{n=-\infty }^\infty n^2 (-x_r)^{n^2}}{\sum _{n=-\infty }^\infty (-x_r)^{n^2}} \right) \left( \sum _{n=-\infty }^\infty x_r^{n^2} \right) ^{-4}, \end{aligned}$$

where $x_r:=q_2(i \sqrt{r})=e^{-\pi \sqrt{r}}$. As *r* tends to infinity, $x_r$ tends to zero and $\alpha (r)$ tends to $1/\pi$. A crucial key in the work of the Borweins is to calculate $\alpha (N)$ for $N\in \mathbb Z_{>0}$ which is done iteratively.

Ramanujan’s series (1) is obtained from Theorem 4.4 by setting $N=58$.

It is interesting to have a glance at the corresponding comment made in [6], “For $N=58$ [$\dots$] Ramanujan [23] and Weber [26] have calculated $g_{58}$ for us [$\dots$] sophisticated number-theoretic techniques exist for computing $k_N$ [$\dots$] Knowing $\alpha$ is equivalent to determining that the number 1103 [in Ramanujan’s series (1)] is correct. It is less clear how one explicitly calculates $\alpha (58)$ in algebraic form, except by brute force, and a considerable amount of brute force is required; but a numerical calculation to any reasonable accuracy is easily obtained from (88) and 1103 appears!”

To complete the gap in proving their derivation of Ramanujan’s series (1) (i.e., proving that 1103 is indeed correct), the Borweins set up a numerical estimation in way that Gosper’s computation delivered sufficiently many digits of $\pi$ to conclude that 1103 is indeed correct. The interested reader finds details of the Borwein reasoning in [6, sec. 8].

In contrast, the Sato construction as described above works purely on algebraic grounds. In other words, if the computational complexity of the problem allows that the required singular values $h_N:=h(\tau _N)$, $p_1(h_N)$, and $p_2(h_N)$ can be computed by a computer algebra implementation of the MultiSamba algorithm, this at the same time is a guarantee for their correctness!

It is even possible to automate the Sato construction with regard to families of Sato triples. For example, for the Sato family (80), the values for $N\ge 2$ where $1/h(i \sqrt{2N})$ is an integer are:

$$ N=2,3,5,9,11,29. $$

Hemmecke’s implementation in FriCAS delivers for all the primes among these values the corresponding series for $1/\pi$; see

https://www.risc.jku.at/people/hemmecke/papers/oneoverpi

### Remark 4.5

Hemmecke is working on an extension of his MultiSamba implementation from primes to arbitrary integers. This then will allow to treat also the case $N=9$, and also the case $N=95$ of the Sato family in (80) which then would deliver a computer proof of $\lim _{m \rightarrow \infty } \phi (m)=1/\pi$ for the approximating sum $\phi (m)$ from (85).

## Sato triples and Chudnovsky families

In this section we present further infinite families of Sato triples where we take as the starting point, Step 0, of the Sato construction the pair

89 $$\begin{aligned} g(\tau )&:= \sqrt{E_4(\tau )}= 1 + 120 q - 6120 q^2 + 737760 q^3 +O(q^4), \end{aligned}$$

90 $$\begin{aligned} h(\tau )&:= \frac{1}{j(\tau )}= q - 744 q^2 + 356652 q^3 +O(q^4), \end{aligned}$$

where $q=q_1(\tau )=e^{2 \pi i \tau }$. Here $E_4(\tau )$ is the normalized Eisenstein series of weight 4, and $j(\tau )$ is the modular j-function (Felix Klein’s j-invariant); i.e., we have

91 $$\begin{aligned} E_4(\tau )\in M_4(\Gamma ) \,\text { and }\, j(\tau )\in M_0(\Gamma ) \,\text { for }\, \Gamma :=\textrm{SL}_2(\mathbb {Z}). \end{aligned}$$

As explained in [21, Sec. 5.1], the following local expansion $(g,h,Y;\Gamma )$ can be determined algorithmically:

92 $$\begin{aligned} Y(z):= \sum _{n=0}^\infty \underbrace{\frac{ (1/6)_n(1/2)_n (5/6)_n}{(1)_n (1)_n n!} 1728^{n}}_{=c(n)} \cdot z^n = \sum _{n=0}^\infty \frac{(6n)!}{(3n)!(n!)^3} z^n \end{aligned}$$

with holonomic coefficient sequence (*c*(*n*)), such that for $\tau$ with $\Im (\tau )$ sufficiently large,

$$ g(\tau )=Y(h(\tau )). $$

### Remark 5.1

Here, as well as for other applications, we need to consider a slightly more general setting for local expansions. Namely, when *g* is defined as a root of a modular form,

$$ g(\tau ):=f(\tau )^{1/r} $$

where $f\in M_{2 r}(\Gamma )$ such that $f(\infty )\ne 0$. It turns out that also in this case a local expansion $(g,h,Y; \Gamma )$ exists with *Y* being again a power series with holonomic coefficients. As indicated in Sect. 10, such local expansions come from the existence of differential equations such as Out[86]. Yifan Yang’s proof  [31, Thm. 1] covers the special case $r=1$. The proof for $g=f^{1/r}$ with $f\in M_{k r }(\Gamma )$, $k\in \mathbb Z_{\ge 1}$, is essentially the same.

Two infinite families of Sato triples for $\Gamma =\textrm{SL}_2(\mathbb {Z})$ give rise to two infinite families of series for $1/\pi$.

### Chudnovsky family 1

The following lemma in a special case gives rise to the celebrated series (87).

#### Lemma 5.2

For each $N\in \mathbb Z_{\ge 2}$ one has that

93 $$\begin{aligned} (N, \gamma _N, \tau _N) = \left( N, \left( \begin{matrix} -1& -1\\ 1& 0 \end{matrix} \right) , \frac{-1+i\sqrt{4N-1}}{2N} \right) \end{aligned}$$

is a Sato triple for $\Gamma =\textrm{SL}_2(\mathbb {Z})$. Moreover,

94 $$\begin{aligned} \tau _N=\left( \begin{matrix} 0& -1\\ 1& 0 \end{matrix} \right) \frac{1+ i \sqrt{4 N-1}}{2}. \end{aligned}$$

#### Proof

The proof is by straight-forward verification. $\square$

Let us choose the local expansion $(g,h,Y; \Gamma )$ with (89), (90), and (92) as the starting point of the Sato construction. Then (93) gives rise to an infinite family of series for $1/\pi$. Note that by (94),

$$ j(\tau _N)=j\left( \frac{1+i \sqrt{4N-1}}{2} \right) $$

which evaluates to cubes of integers in case that $4N-1$ is a Heegner number. The largest one is obtained with $N=41$ for which

$$ j\left( \frac{1+i \sqrt{163}}{2} \right) = - 640320^3, $$

and the corresponding series obtained by the Sato construction is (87).

To present one more member of this family, for $N=17$ one has

$$ j\left( \frac{1+i \sqrt{67}}{2} \right) = - 5280^3 $$

and obtains

95 $$\begin{aligned} \frac{1760 \sqrt{330}}{\pi } = \sum _{n=0}^\infty (10177 + 261702 n) \frac{(1/6)_n(1/2)_n (5/6)_n}{(1)_n (1)_n n!} 1728^{n} \Big (\frac{-1}{5280}\Big )^{3n} \end{aligned}$$

as the result of the Sato construction. Each summand adds about the same number of correct digits to $1/\pi$ as the series (1).

### Chudnovsky family 2

The following lemma, which is straight-forward to prove, presents another elementary family of Sato triples which induces another infinite “Chudnovsky family” of series for $1/\pi$.

#### Lemma 5.3

For each $N\in \mathbb Z_{\ge 2}$ one has that

96 $$\begin{aligned} (N, \gamma _N, \tau _N) = \left( N, \left( \begin{matrix} 0& -1\\ 1& 0 \end{matrix} \right) , \frac{i\sqrt{N}}{N} \right) \end{aligned}$$

is a Sato triple for $\Gamma =\textrm{SL}_2(\mathbb {Z})$.

This means, for $\tau _N$ as in (96),

97 $$\begin{aligned} \tau _N=\left( \begin{matrix} 0& -1\\ 1& 0 \end{matrix} \right) i \sqrt{N}. \end{aligned}$$

Again we choose the local expansion $(g,h,Y; \Gamma )$ with (89), (90), and (92) as the starting point of the Sato construction. Then (96) gives rise to another infinite family of series for $1/\pi$. Note that by (97),

$$ j(\tau _N)=j(i \sqrt{N}), $$

which is an algebraic integer. At particular points one obtains integer values; for example [13, Table (12.20)],

$$ j(i \sqrt{7})= 255^3. $$

Carrying out the Sato construction for the Sato triple with $N=7$, one obtains,

98 $$\begin{aligned} \frac{1}{\pi } = \frac{18 \sqrt{255}}{7225} \sum _{n=0}^\infty (133 n +8) c(n) \Big (\frac{1}{255}\Big )^{3n}, \end{aligned}$$

which was already recorded by Ramanujan [23].

As in the case of the Ramanujan-Gosper family (80), the Sato construction with input (89), (90) and (92), and the infinite families of Sato triples (93) and (96) can be viewed as an algorithmic version of a theorem:

#### Theorem 5.4

([10, Eq. (1.4)] and [11, Eq. (4.9)]) Let

$$ s_2(\tau ):= \frac{E_4(\tau )}{E_6(\tau )} \left( E_2(\tau )-\frac{3}{\pi \Im (\tau )} \right) $$

where the $E_{2j}(\tau )$ are the Eisenstein series as in [28, eqs. (11), (12), (13)]. Then for $\tau =(1+\sqrt{-d})/2$:

99 $$\begin{aligned} \sum _{n=0}^\infty \left( \frac{1-s_2(\tau )}{6} +n\right) \cdot \frac{(6n)!}{(3n)!(n!)^3}\cdot \frac{1}{j(\tau )^n} = \frac{1}{\pi }\cdot \frac{(-j(\tau ))^{1/2}}{(d(1728-j(\tau ))^{1/2}}. \end{aligned}$$

The choice $d=163$ gives (87), a member of the first Chudnovsky family induced by (93).

Despite the fact that the Chudnovskys explicitly restricted^1^ the validity of (99) to points of the form $\tau =(1+\sqrt{-d})/2$, with a mild modification their formula seems to hold also for the second Chudnovsky family induced by (96).

This modification is as follows: instead of restricting to $\tau =(1+\sqrt{-d})/2$, one extends the domain of validity of (99) to all $\tau \in \mathbb H$ which are irrational quadratic numbers with negative discriminant $-d$. For example, the discriminant of $\tau _{41}=(-1+i\sqrt{163})/82$ is $-163$ since $\tau _{41}$ is a root of $41 x^2 + x + 1$ which has discriminant $-163$. The discriminant of $(1+i\sqrt{163})/2$ being a root of $x^2 - x + 41$ is also $-163$. As an example for the suggested extension: the discriminant of $\tau :=i\sqrt{7}$ is $-28$; substituting this $\tau$ into (99) with $d=28$ gives (98), a series of the second Chudnovsky family!

Also for the Chudnovsky families 1 and 2 it is possible to automate the Sato construction; see

https://www.risc.jku.at/people/hemmecke/papers/oneoverpi

for corresponding $1/\pi$ series delivered by Hemmecke’s FriCAS implementation.

## Sato triples and Apéry–Beukers–Chan families

The infinite family of Sato triples presented in this Section in a more direct way relates to the original work of Sato or, more precisely, to the work of Heng Huat Chan and collaborators [9] inspired by Sato.

As the starting point, Step 0, of the Sato construction we take the pair

100 $$\begin{aligned} g(\tau )&:= \prod _{n=1}^\infty \frac{(1-q^{2n})^7 (1-q^{3n})^7}{(1-q^{n})^5 (1-q^{6n})^5} =\frac{\eta (2\tau )^{7}\eta (3\tau )^{7}}{ \eta (\tau )^{5}\eta (6\tau )^{5}}, \end{aligned}$$

101 $$\begin{aligned} h(\tau )&:= q\prod _{n=1}^\infty \frac{(1-q^{n})^{12} (1-q^{6n})^{12}}{(1-q^{2n})^{12} (1-q^{3n})^{12}} =\frac{\eta (\tau )^{12}\eta (6\tau )^{12}}{ \eta (2\tau )^{12}\eta (3\tau )^{12}}, \end{aligned}$$

where $q=q_1(\tau )=e^{2 \pi i \tau }$. We have

102 $$\begin{aligned} g(\tau )\in M_2(\Gamma ) \,\text { and }\, h(\tau )\in M_0(\Gamma ) \,\text { for }\, \Gamma :=\Gamma _1(6). \end{aligned}$$

As explained in [21, Sec. 5.2], this pair is taken from Beukers’ modular-form-based proof [3] of the irrationality of $\zeta (3)$. The following local expansion $(g,h,Y;\Gamma )$ can be determined algorithmically [21, Out[41]]:

103 $$\begin{aligned} Y(z):= \sum _{n=0}^\infty \underbrace{\sum _{k=0}^n \left( {\begin{array}{c}n\\ k\end{array}}\right) ^2 \left( {\begin{array}{c}n+k\\ k\end{array}}\right) ^2}_{=c(n)} \cdot z^n \end{aligned}$$

with holonomic coefficient sequence (*c*(*n*)), such that for $\tau$ with $\Im (\tau )$ sufficiently large,

$$ g(\tau )=Y(h(\tau )). $$

### Remark 6.1

This time the elements *c*(*n*) of the holonomic coefficient sequence are the celebrated Apéry numbers which satisfy a linear recurrence of order two [21, Out[41]]. As stated by Beukers [3, p. 274], the function

$$ y(\tau ):= \frac{\eta (\tau )^{4}\eta (6\tau )^{8}}{ \eta (2\tau )^{8}\eta (3\tau )^{4}}\in M_0(\Gamma ) $$

is a Hauptmodul for $\Gamma =\Gamma _1(6)$; i.e., it generates the field of modular functions on $\Gamma _1(6)$. For example [3, Sec. 2, Eq. (1)],

104 $$\begin{aligned} h(\tau )=y(\tau )\frac{1-9 y(\tau )}{1-y(\tau )}, \end{aligned}$$

which can be found and proven algorithmically as described in Sect. 12.3 using the procedure GuessAE.

### Apéry–Beukers–Chan family 1

This time the *N* in the infinite family of Sato triples comes in parameterized form.

#### Lemma 6.2

For $\ell \in \mathbb Z_{\ge 1}$ one has that

105 $$\begin{aligned} (N, \gamma _N, \tau _N) = \left( 6\ell +1, \left( \begin{matrix} -6\ell +1& -\ell \\ 6& 1 \end{matrix} \right) , \frac{-6\ell +i \sqrt{6 \ell }}{6(6\ell +1)} \right) \end{aligned}$$

is a Sato triple for $\Gamma =\Gamma _1(6)$. Moreover, for $N=6\ell +1$,

106 $$\begin{aligned} \tau _N=\left( \begin{matrix} 1& 0\\ -6& 1 \end{matrix} \right) i \sqrt{\ell /6}. \end{aligned}$$

#### Proof

The proof is by straight-forward verification. $\square$

Consequently, $N=7$ implied by $\ell =1$ will give the first series for $1/\pi$ as the result of the Sato construction based on the local expansion $(g,h,Y; \Gamma =\Gamma _1(6))$ with (100), (101), and (103) and the family (105) of Sato triples. Beukers [3, Prop. 2.1], using the relation (104), proved that

107 $$\begin{aligned} h\left( \frac{i}{\sqrt{6}}\right) = (\sqrt{2}-1)^4. \end{aligned}$$

However, for this choice the series $Y\left( (\sqrt{2}-1)^4 \right)$ does not converge.

Hence the next candidate value from Lemma 6.2 is $N=13$, implied by the choice $\ell =2$. A closed form of the corresponding image $h_2:=h(i/\sqrt{3})$ is easily guessed algorithmically. We start with a numerical approximation;the first 10 digits will be sufficient: 
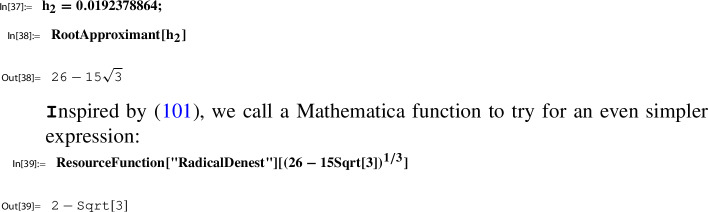
 Summarizing, Mathematica suggests the value

108 $$\begin{aligned} h\left( \frac{i}{\sqrt{3}} \right) = (2-\sqrt{3})^3. \end{aligned}$$

The corresponding series for $1/\pi$ is

109 $$\begin{aligned} \frac{1}{\pi }= \frac{6}{9+\sqrt{3}}\sum _{n=0}^\infty \left( 2n+1-\frac{1}{\sqrt{3}} \right) c(n) (2-\sqrt{3})^{3n}, \end{aligned}$$

where the *c*(*n*) are the Apéry numbers from (103).

This series is the first instance $\ell =2$ of an infinite family specified in a theorem by H.H. Chan and H. Verrill. It involves *h* as in (101), and an entity $p_g$ whose non-trivial definition can be found in their paper.

#### Theorem 6.3

([18, Eq. (5.1)]) Let $t_0:=h(i/\sqrt{6 \ell })$. Then for $\ell \in \mathbb Z_{\ge 2}$,

110 $$\begin{aligned} \frac{1}{\pi } = \frac{\sqrt{\ell }\sqrt{t_0^2-34 t_0+1}}{\sqrt{6}}\sum _{n=0}^\infty \left( 2n+p_g \right) c(n) t_0^n, \end{aligned}$$

where the *c*(*n*) are the Apéry numbers from (103).

#### Remark 6.4

Note that the invariance [3, p. 274], $h(-1/(6\tau ))=h(\tau )$, implies

$$ h\left( i \sqrt{\frac{\ell }{6}} \right) = h\left( i \frac{1}{\sqrt{6 \ell }} \right) . $$

As noted by Chan and Verrill, the instance $\ell =5$ (i.e., $N=31$ in (105)) recovers Sato’s original example [24, entry 1, p. 2],

111 $$\begin{aligned} \frac{1}{\pi }= \frac{4 \sqrt{15}}{9+4\sqrt{5}}\sum _{n=0}^\infty \left( 2n+1-\frac{3\sqrt{5}}{10} \right) c(n) (2-\sqrt{5})^{4n}, \end{aligned}$$

where the *c*(*n*) are the Apéry numbers from (103).

### Apéry–Beukers family 2

We conclude this section with another infinite family of Sato triples different from (105). Despite the similar parameterization, it gives rise to a family of series for $1/\pi$ different from (110) but still involving the Apéry numbers in the coefficient sequences.

#### Lemma 6.5

For $\ell \in \mathbb Z_{\ge 1}$ one has that

112 $$\begin{aligned} (N, \gamma _N, \tau _N) = \left( 6\ell +1, \left( \begin{matrix} -6 \ell -5& -\ell -1\\ 6& 1 \end{matrix} \right) , \frac{-6\ell -3+i \sqrt{6\ell -3}}{6(6\ell +1)} \right) \end{aligned}$$

is a Sato triple for $\Gamma =\Gamma _1(6)$. Moreover, for $N=6\ell +1$,

113 $$\begin{aligned} \tau _N=\left( \begin{matrix} 1& 0\\ -6& 1 \end{matrix} \right) \left( \frac{1}{2}+\frac{i}{2} \sqrt{{2\ell -1\over 3}} \right) . \end{aligned}$$

#### Proof

The proof is by straight-forward verification. $\square$

Taking this family of Sato triples together with the local expansion $(g,h,Y; \Gamma =\Gamma _1(6))$ with (100), (101), and (103) as input for the Sato construction gives a family of series for $1/\pi$ different from (110). The first five relevant *g* values are:

$$\begin{aligned}&g(\tau _{7})=-1,\,\, g(\tau _{13})=-(2 - \sqrt{3})^2,\,\, g(\tau _{19})= -\left( \frac{3 - \sqrt{5}}{2} \right) ^4,\\&g(\tau _{25})= \left( \frac{\sqrt{21}-5}{2} \right) ^3,\,\, g(\tau _{31})=-\left( -1 + 2^{1/3} \right) ^4. \end{aligned}$$

We restrict ourselves to state only one of the corresponding series for $1/\pi$; for the Sato triple (113) with $N=25$, implied by $\ell =4$, the Sato construction results in,

114 $$\begin{aligned} \frac{1}{\pi } = \frac{21\sqrt{3}}{14 +9\sqrt{21}} \sum _{n=0}^\infty \Big ( 4n+2 -\frac{5\sqrt{21}}{21} \Big ) c(n) \left( \frac{\sqrt{21}-5}{2} \right) ^{3n}, \end{aligned}$$

where the *c*(*n*) are the Apéry numbers from (103).

#### Remark 6.6

At the time of the completion of the paper, Ralf Hemmecke started to work on an implementation of our method to automatically derive series for $1/\pi$ induced by Sato triples of the Apéry-Beukers families.

## The algorithm MultiSamba illustrated

The algorithmic aspects of the Sato construction, as described above, involves the following algorithmic aspects:

(i) the holonomic toolbox, primarily for guessing but also for proving;
(ii) the algorithm ModFormDE for proving the correctness of a conjectured linear differential equation in derivatives of a modular form and with coefficients being polynomials in a modular function;
(iii) the main computational engine, the algorithm MultiSamba to derive and prove algebraic relations between modular functions.

Further information on aspects (i) and (ii) can be found in [20] and [21]. A description of the functionality of the algorithm MultiSamba is the object of this section. We restrict to presenting illustrating examples. A more detailed description can be found in [17].

### Working with expansions at the cusps

When dealing with modular functions being analytic on $\mathbb H$, the MultiSamba algorithm works by representing them in the form of tuples of Fourier series expansions at the cusps.

#### Example 7.1

Recall the definition of the modular function $\lambda (\tau )\in M_0(\Gamma (2))$ from (20). For MultiSamba it is represented by a triple,

$$ \lambda \leftrightarrow (\lambda _\infty , \lambda _0, \lambda _1), $$

where the entry $\lambda _r$ is a truncated Fourier series representation of $\lambda$ at the cusp *r*.

The representation by triples in the example is owing to the fact that the group $\Gamma =\Gamma (2)$ induces the three cusps $\infty , 0$, and 1. In general, if MultiSamba has to deal with modular functions in $M_0(\Gamma )$ where $\Gamma$ induces *n* cusps, the functions are represented by *n*-tuples. The word “Multi” in MultiSamba stands for doing computations with such tuples, in this way extending the algorithm Samba (“subalgebra module basis algorithm”) originating from [22] and [16], which works with standard Laurent series (in the Fourier variable).

The required order of truncation depends on the particular application. In the computer algebra system FriCAS, there is no need to precompute a truncation point, because it provides a lazy Laurent series implementation that computes coefficients at the time they are needed for the concrete computation.

#### Example 7.2

TASK. Prove algorithmically the classical relation (122) occuring in Sect. 8,

115 $$\begin{aligned} \frac{1}{j(\tau )} =\frac{(\lambda (\tau )-1)^2 \lambda (\tau )^2}{256 \left( \lambda (\tau )^2-\lambda (\tau )+1\right) ^3}. \end{aligned}$$

The functions $j(\tau )$ and $\lambda (\tau )$ are analytic on $\mathbb H$. Hence the proof of (115) reduces to the verification of the relation rel=0 at the cusps, where 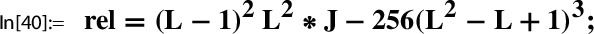 Here *J* and *L* are symbols standing for the *j* and $\lambda$ function, respectively. Next we input suitable representations of $\lambda$ and *j* in terms of triples

$$ \lambda \leftrightarrow (\lambda _\infty , \lambda _0, \lambda _1) \,\text { and }\, j\leftrightarrow (j_\infty , j_0, j_1), $$

consisting of expansions at the cusps: 
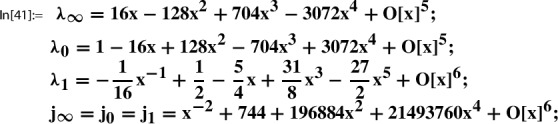
 the symbol *x* stands for $x=q_2=e^{\pi i \tau }$.

#### Remark 7.3

In Hemmecke’s FriCAS toolbox the expansions from In[41] are computed with the package QEta [15].

As operations on lists (recall: Mathematica uses “$\{\dots \}$” notation for lists), addition and multiplication are carried out componentwise; for example, 
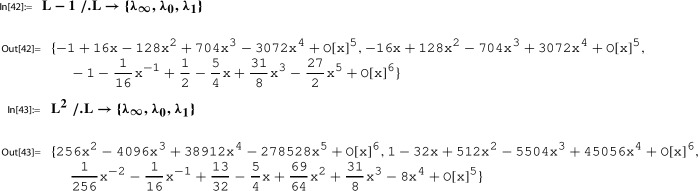
 This arithmetical feature of Mathematica makes the verification of rel=0 particularly convenient: 
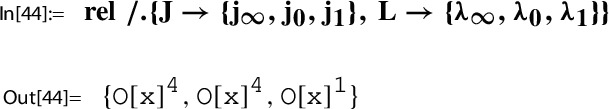
 The meaning of this output list is this: in each of its three components the corresponding Laurent series involves no summand with non-negative power of *x*. In other words, this verifies that at each cusp the modular function

$$ (\lambda (\tau )-1)^2 \lambda (\tau )^2 \cdot j(\tau ) -256 \left( \lambda (\tau )^2-\lambda (\tau )+1\right) ^3, $$

which corresponds to the symbolic expression rel, has no pole and has no non-zero constant part in its *x*-expansion. This implies that it is zero, and (115) is proven.

#### Remark 7.4

If one would work with a description of *j* with smaller trunctation order, e.g., 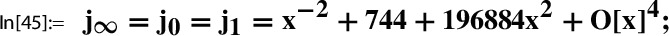 the output triple would contain a constant (unspecified) or *x*-powers of negative order. For example, in the concrete case of input In[45] one has, 
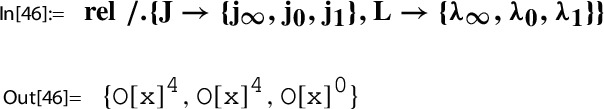
 which would not suffice for a proof of rel=0. In other words, the user has to take care of choosing truncation orders of sufficient size.

### Computing algebraic relations

Given modular functions $\phi , \psi \in M_ 0(\Gamma )$ which are analytic on $\mathbb H$, in this section we informally describe how the algorithm MultiSamba computes a polynomial *p*(*X*, *Y*), with coefficients in a suitable extension field $\mathbb K$ of $\mathbb Q$, such that $p(\phi (\tau ),\psi (\tau ))=0$.

As explained in the previous Sect. 7.1, instead of working directly with the functions $\phi$ and $\psi$, MultiSamba operates on representatives which are *n*-tuples of formal Laurent series in *x*, *n* being the number of cusps induced by $\Gamma$. Recall that analytically $x=q_w=e^{2 \pi i \tau /w}$, but owing to a problem transformation onto algebraic grounds, MultiSamba interprets *x* as a symbolic variable.

To compute *p*(*X*, *Y*), MultiSamba proceeds by using a *reduction* mechanism: Let $f=(f_1,\dots ,f_n)$ be an *n*-tuple of formal Laurent series in *x*. Let $B=\{\textbf{1},b_0, b_1,....,b_k\}$ be a finite set of *n*-tuples, each $b_j$ being a formal Laurent series in *x* and $\textbf{1}$ standing for the *n*-tuple $(1,\dots ,1)$. A *reduction* of *f* with respect to *B* is an *n*-tuple $(g_1,\dots , g_n)$ defined as

116 $$\begin{aligned} (g_1,\dots , g_n):= f-(\alpha \textbf{1}+\beta _0\, b_0+\dots +\beta _k\, b_k) \end{aligned}$$

with $$\alpha , \beta _j\in \mathbb K$$.

The notion of *reduction* is justified by using a special order relation,

117 $$\begin{aligned} (f_1,\dots ,f_n) \succeq (g_1,\dots ,g_n). \end{aligned}$$

Its precise specification is of quite technical nature; it is presented in detail in [17].

#### Remark 7.5

A more elementary reduction relation would be to define (117) whenever the rightmost non-zero entry in

$$ ({{\,\textrm{ord}\,}}f_1 - {{\,\textrm{ord}\,}}g_1, {{\,\textrm{ord}\,}}f_2 - {{\,\textrm{ord}\,}}g_2, \dots , {{\,\textrm{ord}\,}}f_n - {{\,\textrm{ord}\,}}g_n) $$

is negative (“reverse lexicographic ordering”). This “$\succeq$” reduction would indeed work for the next Example 7.6, which can be easily checked in each particular step. But, as already indicated, the reduction to make MultiSamba work in general is more complicated and requires also division by the order of $b_0$, kind of a special element in the reduction process; in the Example 7.6, $b_0=J=(j_\infty ,j_0,j_1)$ and since $({{\,\textrm{ord}\,}}j_\infty , {{\,\textrm{ord}\,}}j_0, {{\,\textrm{ord}\,}}j_1)= (-2,-2,-2)$ (equal entries), division can be avoided. The actual ‘$\succeq$” used my MultiSamba looks at the component that contains the smallest order (breaking ties by selecting bigger components first). Hence “reverse lexicographic order” in general is not applicable.

#### Example 7.6

We use the MultiSamba algorithm to derive the algebraic relation,

118 $$\begin{aligned} {(\lambda (\tau )-1)^2 \lambda (\tau )^2}\cdot {j(\tau )}- {256 \left( \lambda (\tau )^2-\lambda (\tau )+1\right) ^3}=0, \end{aligned}$$

which is equivalent to (115).

Corresponding to In[41] of Example 7.2, we first input triples *L* and *J* as representatives of $\lambda$ and *j*, respectively. 
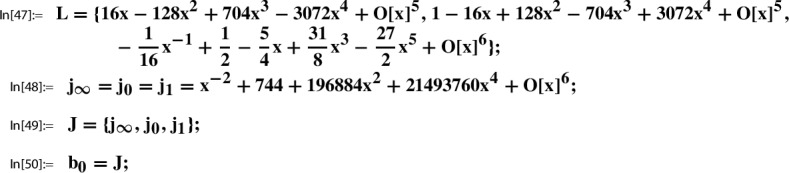
 As components of *L* we chose again the $\lambda _r$ from In[41].

In the last line we defined $b_0:=J$. Trying to reduce *L* with respect to $\{1,b_0\}$ fails, which is obvious in view of ${{\,\textrm{ord}\,}}j_1 = -2$ in contrast to ${{\,\textrm{ord}\,}}l_1 = -1$.

As a repeated step of the algorithm, we take the result of the reduction, which in this case is *L* itself, as an additional reduction element $b_1$, 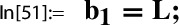 Then we try to reduce $L\cdot b_1$ w.r.t. $\{1,b_0,b_1\}$: 
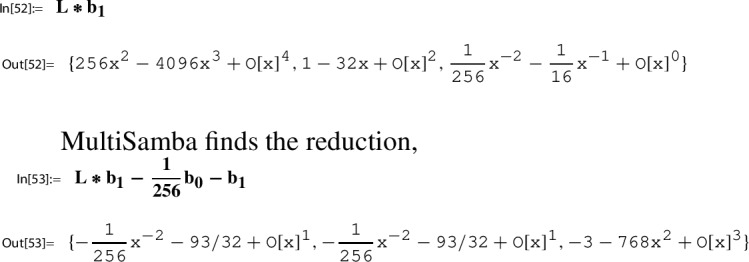
 Again we take the result of the reduction as an additional reduction element $b_2$, 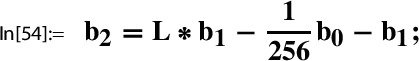 Next we try to reduce $L\cdot b_2$ w.r.t. $\{1,b_0,b_1,b_2\}$: 
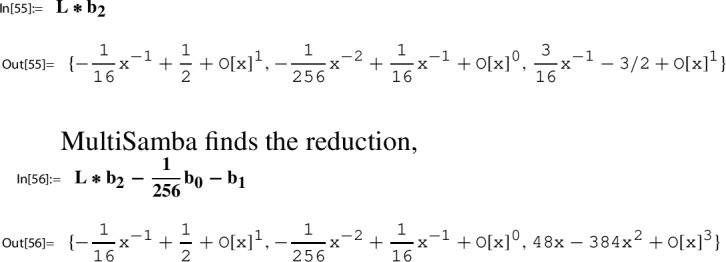
 Again we take the result of the reduction as an additional reduction element $b_3$, 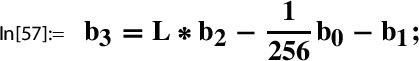 Then we try to reduce $L\cdot b_3$ w.r.t. $\{1,b_0,b_1,b_2,b_3\}$. This reduction fails, and we take the result of the reduction, which is $L\cdot b_3$ itself, as an additional reduction element $b_4$, 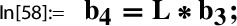 Next we try to reduce $L\cdot b_4$ w.r.t. $\{1,b_0,b_1,b_2,b_3,b_4\}$: 
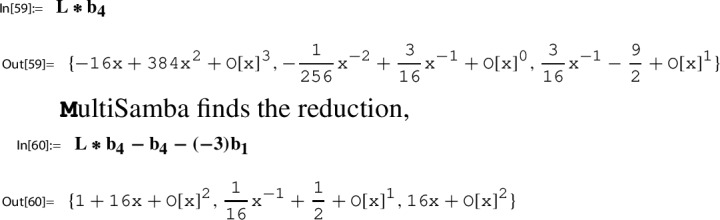
 Again we take the result of the reduction as an additional reduction element $b_5$, 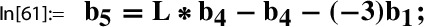 Next we try to reduce $L\cdot b_5$ w.r.t. $\{1,b_0,b_1,b_2,b_3,b_4,b_5\}$: 
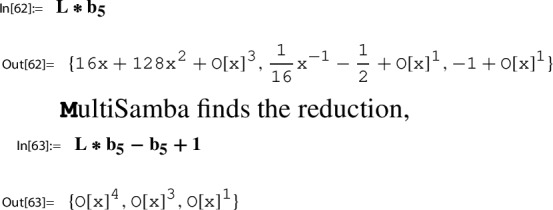
 This means,

$$ 0= L\, b_5-b_5+\textbf{1}. $$

With backwards substitution one obtains,

$$\begin{aligned} 0&= L\, b_5-b_5+\textbf{1}=(L -\textbf{1}) b_5 +\textbf{1}\\&= (L-\textbf{1})\left( (L-\textbf{1})b_4- (-3) b_1\right) +\textbf{1}\\&= (L-\textbf{1})\left( (L-\textbf{1})L\, b_3+3 b_1\right) +\textbf{1}\\&= (L-\textbf{1})\left( (L-\textbf{1})L\, (L\, b_2 +3 b_1) +3 b_1\right) +\textbf{1}\\&= (L-\textbf{1})^2 L^2 b_2+ 3(L-\textbf{1})(L^2-L+\textbf{1})b_1 +\textbf{1}\\&= (L-\textbf{1})^2 L^2 \left( L\, b_1 -b_1- \frac{1}{256} b_0\right) + 3(L-\textbf{1})(L^2-L+\textbf{1})b_1 +\textbf{1}\\&= -\frac{1}{256}(L-\textbf{1})^2 L^2 b_0 + \left( (L-\textbf{1})^3 L^2 b_1 + 3(L-\textbf{1})(L^2-L+\textbf{1})b_1 +\textbf{1} \right) \\&= - \frac{1}{256}(L-\textbf{1})^2 L^2 \cdot J + \left( (L-\textbf{1})^3 L^3+ 3(L-\textbf{1})(L^2-L+\textbf{1})L +\textbf{1} \right) . \end{aligned}$$

Observing that

$$ (L-\textbf{1})^3\,L^3+ 3(L-\textbf{1})(L^2-L+\textbf{1})L +\textbf{1}= (L^2-L+\textbf{1})^3, $$

implies

119 $$\begin{aligned} p(X,Y)= -\frac{1}{256}(X-1)^2 X^2 \cdot Y +(X^2-X+1)^3, \end{aligned}$$

which completes the derivation of (118).

We conclude this section with a couple of remarks. First, once an algebraic relation is derived with MultiSamba, this derivation stands also for a correctness proof of this relation.

Second, in the reduction process of Example 7.6 the variable $b_0=J$ arises in only one reduction relation, In[53]; hence *Y* occurs only linearly in (119). This is owing to the fact that $\lambda$ is a Hauptmodul for $\Gamma (2)$. In general, the variable $b_0$ will arise in more than one reduction relation and thus *Y* will occur non-linearly in the output polynomial *p*(*X*, *Y*).

Finally, the proof that one can specify an order relation (117) such that MultiSamba reduction works and terminates also in general is presented in [17].

## Proof of $h(\tau _{29})=1/396^4$ and related computational aspects

An immediate proof of (54) is obtained by substituting the value

120 $$\begin{aligned} \lambda (i \sqrt{58})= (13\sqrt{58}-99)^2(\sqrt{2}-1)^{12}, \end{aligned}$$

entry (33) of [29], into the definition (50) of $\lambda$.

At the end of Sect. 3.7 we indicated a hybrid numeric-symbolic strategy for closed form evaluation of $h(\tau _N)$. To obtain an algebraic form of $\lambda (i \sqrt{58})$, one could, for example, proceed as follows. Again we use the Mathematica built-in function ModularLambda for modular $\lambda$. In the first step we compute the desired value with a 100-digit precision. 
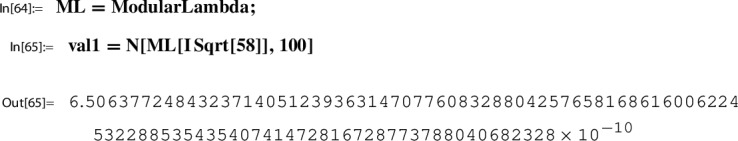
 Next we use a Mathematica procedure which suggests a polynomial having a root located “as close as possible” to the numerical approximation val1 of $\lambda (i \sqrt{58})$: 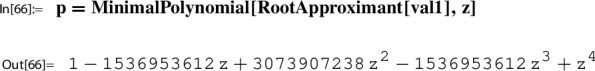 The built-in solver computes four roots represented in terms of nested radicals. Out of those we pick that one which is equal to the expression on the right of (120): 
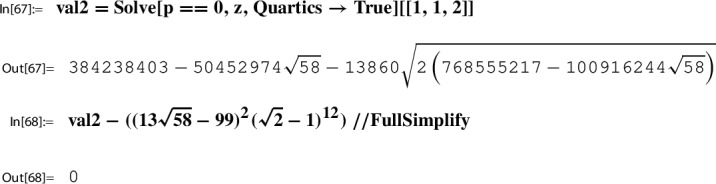
 We remark that there is a Mathematica function which tries to “denest” nested radicals: 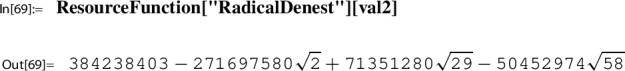


A correctness proof of the root representation val2 can be obtained by using the classical evaluation for the modular *j* function,

121 $$\begin{aligned} j(i \sqrt{58})= 30^3(140989+26163 \sqrt{29})^3, \end{aligned}$$

entry (58) of [30], together with the classical relation

122 $$\begin{aligned} j(\tau )=2^8 \frac{(1-\lambda (\tau )+\lambda (\tau )^2)^3}{\lambda (\tau )^2 (1-\lambda (\tau ))^2}, \end{aligned}$$

entry (2) of [30].

### Remark 8.1

The evaluation (121) is classical in the following sense, as described at [30]. There are 18 numbers *d* having class number $h(-d)=2$, where $h(-d)$ is the class number of the binary quadratic form discriminant $-d$ of the quadratic field $\mathbb Q(\sqrt{-d})$. The evaluation (121) falls into the group of six entries [30, (53) to (59)] where *d* is of the form $d=4m$; the case $m=58$ corresponds to (121).

The computational correctness proof goes as follows: 
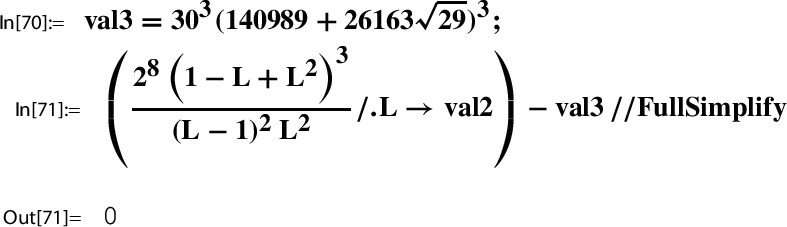


Algorithmic discovery and proof of (122) using the MultiSamba algorithm is discussed in Sect. 7.

Algorithmic discovery of (121) can be done analogously to the finding of an algebraic representation of $\lambda (i \sqrt{58})$ as shown before.

### MultiSamba discovers and proves $h(i \sqrt{58})=1/396^4$

We conclude this section by describing how to derive,

$$ h(\tau _{29})=h(i \sqrt{58}) = \left( \frac{1}{396^4} \right) , $$

with the help of Hemmecke’s implementation of the MultiSamba algorithm.

For our running Example 2.6 we consider the function $\iota (\tau ):=\frac{1}{h(\tau )}$, see (17). It is easy to verify that $\iota (\tau )$ does not have any pole in $\mathbb H$; expansions at the cusps can be easily obtained. The same holds true for the function $\iota (29\tau )$. Analogously to our description in Sect. 9, with the help of MultiSamba we can find a polynomial $f\in \mathbb Z[x,y]$ such that $f(\iota (\tau ),\iota (29\tau ))=0$ for every $\tau \in \mathbb H$. In particular for $\tau _{29}$ as given by (10), we have $\iota (\tau _{29})=\iota (29\tau _{29})$. Therefore, $\iota (\tau _{29})$ must be a root of the polynomial *f*(*x*, *x*). This polynomial is of degree 58 and has the following primitive factors over $\mathbb Q$:

123 $$\begin{aligned} {\begin{matrix} &  x - 24591257856, \quad x - 2509056, \quad x - 2304, \quad x - 648, \quad x - 81, \quad x,\\ &  x + 1024, \quad x + 3969, \quad x + 12288, \quad x + 82944, \quad x + 6635520,\\ &  x^2 - 695296512 x + 691798081536, \quad x^2 - 16584912 x + 69257922561,\\ &  x^2 + 68825088 x + 21743271936, \quad x^2+3553320960 x-770527199232,\\ &  x^2 + 19990130688 x + 52205595918336,\\ &  x^3 - 10618854144 x^2 - 204506726400 x - 19677812097024,\\ &  x^3 + 22252544 x^2 - 11204034560 x + 2578054119424,\\ &  x^4-1265184 x^3+1624487296 x^2-89027692544 x + 7815289901056. \end{matrix}} \end{aligned}$$

Note that according to (42), we have $\iota (\tau _{29})=\iota (i\sqrt{58})$. One can find rational numbers $l_x$ and $u_x$ such that $l_x< x_\infty := e^{- \pi \sqrt{58}} < u_x$; this is done by using upper and lower bounds from $\mathbb Q$ sufficiently close to $\pi \sqrt{58}$, and then truncating the exponential series expansion appropriately.

With the help of these bounds we can find rational upper and lower bounds of $\lambda (i\sqrt{58})$ by using the first few terms of the defining $\theta$-functions according to (20). Then, using (17), we can find rational numbers *l* and *u* such that $l< \iota (i\sqrt{58}) < u$.

By the above construction, it is clear that we can make the interval (*l*, *u*) as small as desired. In particular, we can make it so small that there is only one factor from (123) that has a root in (*l*, *u*). Furthermore, this interval should be made so small that there is only one of the roots of this factor in (*l*, *u*). Checking the number of real roots of a polynomial inside a given interval can be done constructively by applying Sturm’s Theorem [25, Ex. 4.32].

Finally, since $l< \iota (i\sqrt{58}) < u$ and there is exactly one real root of one particular factor from (123) in the interval (*l*, *u*), this root must be equal to $\iota (i\sqrt{58})$. In our case, the interval (*l*, *u*) is such that the first factor from (123) is selected, which eventually leads to $h(\tau _{29}) = \frac{1}{396^4}$.

All the above steps are automated in the QEta package, i.e. the value $h(\tau _N)$ can be found algorithmically from a Sato triple.

## Algorithmic computation of $p_1(h(\tau _N))$ and $p_2(h(\tau _N))$

Using the algorithm MultiSamba one cannot only find but also prove algebraic representations of $p_1(h(\tau _N))$ and $p_2(h(\tau _N))$. In this section we present the MultiSamba method by describing how to derive and prove the closed form representations in (67) of Lemma 3.18 and in (73) of Lemma 3.22. In the following two sections *N* is specified as $N:=29$, but to make the general idea more transparent we keep the generic variable *N* in.

### Computation of $p_1(h(\tau _N))$

The general idea is to derive a low degree polynomial $p\in \mathbb Z[x]$ such that $p_1(h(\tau _N))$ is a root of *p*.

Recall the definition of $g\in M_2(\Gamma ; \chi )$ and $h\in M_0(\Gamma )$ from (76) for the group $\Gamma$ given by (77). Furthermore, by Lemma 3.16 we know $\frac{H(\tau )}{g(\tau )}\in M_0(\Gamma '_\chi )$. We also have $h(\tau ),h(N\tau )\in M_0(\Gamma '_\chi )$. Consequently, there are polynomials $f_1(x,y)$, $f_2(x,y) \in \mathbb Z[x,y]$ such that

$$ f_1\!\left( h(\tau ), \frac{H(\tau )}{g(\tau )} \right) = 0 \,\text { and }\, f_2\!\left( h(N\tau ), \frac{H(\tau )}{g(\tau )} \right) = 0 \,\text { for any }\, \tau \in \mathbb H. $$

By the Sato triple property we have $h(\tau _N)=h(N\tau _N)$. Hence it follows that $\frac{H(\tau _N)}{g(\tau _N)}$ must be a root of the polynomial $p(x):= \gcd (f_1(h(\tau _N), x), f_2(h(\tau _N), x))$.

Our goal is to compute such modular polynomials $f_1$ and $f_2$ using the algorithm MultiSamba which is implemented in FriCAS as part of Hemmecke’s QEta package. In general, such computations are rather technical and time consuming.

Moreover, to bring MultiSamba into position, some preprocessing is required. As explained in Sect. 7, MultiSamba represents a modular function by its expansions at the cusps; in addition, the modular functions in MultiSamba have to be analytic on $\mathbb H$. Consequently, poles in $\mathbb H$ must be removed first. Therefore, we used $\iota (\tau ):= \frac{1}{h(\tau )}$ and $K(\tau ):= \frac{29}{24} \iota (\tau ) \iota (N\tau ) \frac{H(\tau )}{g(\tau )}$ instead of $\frac{H(\tau )}{g(\tau )}$, and find $f_1$ and $f_2$ by computing the modular polynomials $\bar{f}_1,\bar{f}_2$ such that

$$\begin{aligned} \bar{f}_1(\iota (\tau ),K(\tau ))=\bar{f}_2(\iota (N\tau ),K(\tau ))=0, \end{aligned}$$

respectively, using MultiSamba. In fact, we compute

$$\begin{aligned} \bar{p}(x)=\gcd (\bar{f}_1(\iota (\tau _N),x), \bar{f}_2(\iota (\tau _N),x)). \end{aligned}$$

For the particular case of our running Example 2.6 we have $\iota (\tau _N)=396^4$ and get $\deg (\bar{f}_1(396^4,x))=\deg (\bar{f}_2(396^4,x))=60$ and $\deg (\bar{p}(x))=2$. While the monic polynomials $\bar{f}_1(396^4,x), \bar{f}_2(396^4,x)$ have coefficients with up to about 1400 digits, their greatest common divisor is

$$\begin{aligned} \bar{p}(x) = x^2 - 3731029805450945403426466863644562948096. \end{aligned}$$

By taking the positive root and undoing the multiplication by $\iota (\tau _N)\iota (N\tau _N)=396^8$, we obtain $p_1(h(\tau _{29}))=\frac{4412 \sqrt{29}}{284229}$.

For further computational details ; see

https://www.risc.jku.at/people/hemmecke/papers/oneoverpi

### Algorithmic computation of $p_2(h(\tau _N))$

Recall Lemma 3.20 where we proved

$$ \frac{{h'}(\tau )}{g(\tau )} \in M_0(\Gamma _\chi ) \,\text { with }\, \Gamma _\chi :=\Big \{\left( \begin{matrix} a& b\\ c& d \end{matrix} \right) \in \Gamma : \chi ( a, b, c, d) =1 \Big \}\le \Gamma , $$

and $\Gamma$ as in (77). Also, $h(\tau )\in M_0(\Gamma _\chi )$, hence in view of $h(\tau _N)=1/396^4$ we aim at computing a polynomial $f\in \mathbb Z[x,y]$ such that $f(h(\tau ), p_2(h(\tau ))=0$.

As in the previous section, to bring MultiSamba into position, we first remove all poles, again by using $\iota (\tau ):= \frac{1}{h(\tau )}$. Then we invoke MultiSamba to compute instead of *f* the related modular polynomial $\bar{f}$ such that

$$\begin{aligned} \bar{f}(\iota (\tau ),L(\tau ))=0 \,\text { for }\, L(\tau ):=\iota (\tau )^2p_2(h(\tau )). \end{aligned}$$

For our running Example 2.6 it turns out that this polynomial is of degree 2 and equal to $\bar{f}(x,y)=y^2-x(x-256)$. By substituting $x=396^4$, taking the positive root and undoing the multiplication by $\iota (\tau _{29})^2=396^8$, we get $p_2(h(\tau _{29}))=\frac{455\sqrt{29}}{60254729561664}$.

For further computational details ; see

https://www.risc.jku.at/people/hemmecke/papers/oneoverpi

## Algorithmic derivation of local expansion (24)

The algorithmic discovery of local expansions using the holonomic toolbox is described in detail in [21]. To obtain (24), one proceeds as follows.
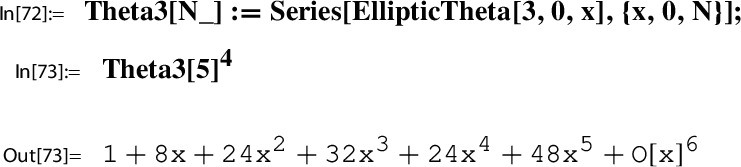
 Instead of invoking Mathematica’s built-in functions such as EllipticTheta, in various cases one may prefer alternative definitions^2^: 
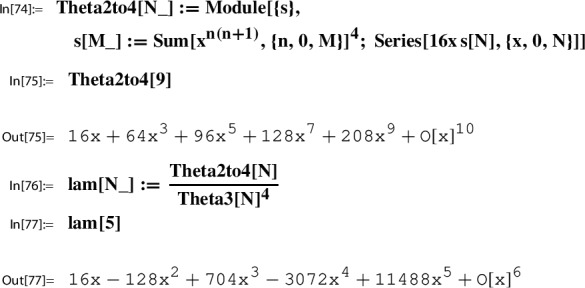
 Next we input the *x*-series for *g* and *h*; see also (21) and (22): 
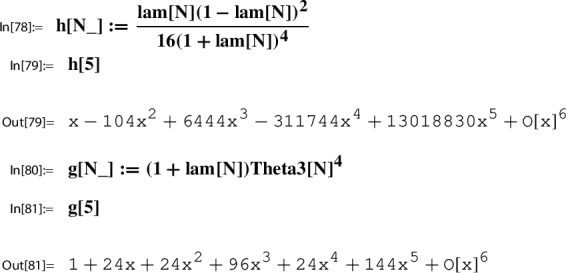


Now we are ready to compute the first twelve coefficients *c*(0) to *c*(11) such that (24): 
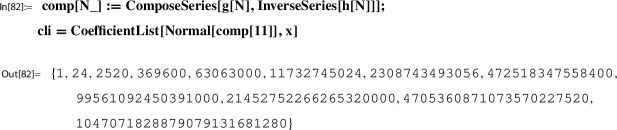


To carry out the next step, we load Mallinger’s package GeneratingFunctions written in Mathematica, which is freely available at https://combinatorics.risc.jku.at/software. This RISC package implements a holonomic-tool box for guessing and proving; see [21] for further details: 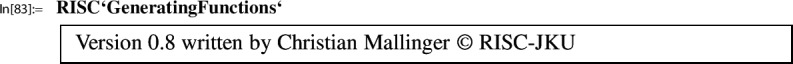


Based on the first twelve values *c*(0) to *c*(11) as input, Mallinger’s procedure GuessRE produces a *guess* of a recurrence the holonomic coefficient sequence $(c(n))_{n\ge 0}$ might satisfy such that (24) holds: 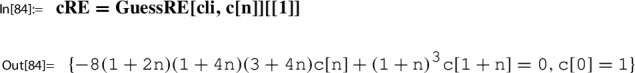


Applying RSolve, a recurrence solver coming with the Mathematica system, produces a closed form for the *c*(*n*): 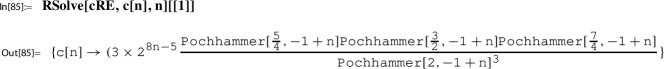 which matches (23).

So far, the recurrence for the *c*(*n*) and their closed form (23) were derived as *conjectures*. But, as explained in [20] and [21], there is an algorithmic method, the algorithm ModFormDE, which delivers a computer-assisted proof for this. This proof is based on holonomic transformation and on zero recognition for modular functions. More concretely, the transformation consists in rewriting the recurrence cRE for the *c*(*n*) into an equivalent form, namely, into a differential equation for the generating function

$$ Y(z)=\sum _{n=0}^\infty c(n) z^n. $$

This step is carried out automatically as follows: 
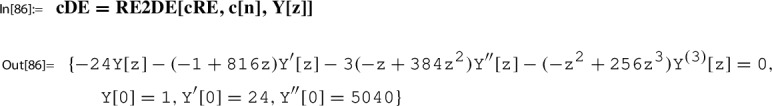


Solving this differential equation with Mathematica’s DSolve, confirms the closed form (23): 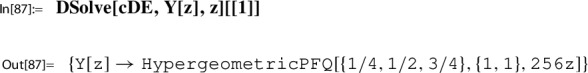 Summarizing, by (4) we know there exists a local expansion such that for all $\tau \in \mathbb H$ with $\Im (\tau )$ sufficiently large,

$$ g(\tau )=Y(h(\tau )) \,\text { with }\, Y(z)=\sum _{n= 0}^\infty c(n) z^n $$

and $(c(n))_{n\ge 0}$ a holonomic sequence. Using the holonomic toolbox we arrived at the conjecture that the *c*(*n*) satisfy the recurrence Out[84]. This conjecture in turn is equivalent to the statement that the generating function *Y*(*z*) satisfies the differential equation Out[86]. As described in [21] and [21] one can prove the validity of such conjectured differential equation algorithmically. The underlying idea in short is this: one takes the differential equation part of Out[86] in the analytic version

$$\begin{aligned}&-24 Y(h(\tau )) - (-1 + 816 h(\tau )) Y'( h(\tau )) - 3 (- h(\tau ) + 384 h(\tau )^2) Y''(h(\tau )\\&\hspace{0.7cm} - (-h(\tau )^2 + 256 h(\tau )^3) Y^{(3)}(h(\tau )) \end{aligned}$$

and, using

$$ Y(h(\tau ))=g(\tau ), Y'(h(\tau ))= \frac{g'(\tau )}{h'(\tau )}, \,\text { a.s.o.}, $$

rewrites it into a form involving *g* and *h* and their derivatives only. Then one rewrites the resulting expression into “Yang form” [20, Lemma 4.7]; i.e., as a linear combination of functions with uniquely determined coefficients being modular functions for $\Gamma$. Finally, showing this linear combination to be zero amounts to algorithmic zero recognition of the modular function coefficients. Details are given in [20].

## Neighborhoods for $g(\tau )=Y(h(\tau ))$

Task (59) of Step 6 of the Sato construction is to determine a bound $L> 0$ such that

$$ g(\tau )=Y(h(\tau )) \,\text { with }\, Y(z)=\sum _{n= 0}^\infty c(n) z^n \,\text { holds for all }\, \Im (\tau )>L. $$

In this section we use our running example to illustrate how this can be done.

For convenience we display the required representations of the functions involved. For $x=e^{\pi i \tau }$,

124 $$\begin{aligned} \theta _2(\tau )= \sum _{n\in \mathbb Z+1/2} x^{n^2} = 2x^{1/4}\sum _{n=0}^{\infty }x^{n^2+n}, \end{aligned}$$

and

125 $$\begin{aligned} \theta _3(\tau )=\sum _{n\in \mathbb Z} x^{n^2} = 1 + 2 \sum _{n=1}^\infty x^{n^2}, \end{aligned}$$

Recall,

$$ g(\tau ):=(1+\lambda (\tau )) \theta _3(\tau )^4 \,\text { and }\, h(\tau ):= \frac{\lambda (\tau ) (1 - \lambda (\tau ))^2}{16 (1 + \lambda (\tau ) )^4}, $$

where

126 $$\begin{aligned} \lambda (\tau )=\frac{\theta _2(\tau )^4}{\theta _3(\tau )^4}. \end{aligned}$$

In (64) we stated a local expansion with neighborhood of validity,

127 $$\begin{aligned} g(\tau )=\sum _{n=0}^\infty \frac{ (1/4)_n(1/2)_n (3/4)_n}{(1)_n (1)_n n!} 256^n h(\tau )^n,\, \, \Im (\tau )>1.87. \end{aligned}$$

In this section we want to prove that one indeed can choose the bound

128 $$\begin{aligned} L:=1.87. \end{aligned}$$

As a first observation, one can quickly see that

129 $$\begin{aligned} L\ge 1. \end{aligned}$$

### Proof of (129)

By inspection, $h(\tau )$ has a pole at $\tau _h:=1+i$ since

$$ \lambda (1+i)=-1; $$

just set $\tau =i$ in the classic transformations

$$ \lambda (\tau +1) =\frac{\lambda (\tau )}{\lambda (\tau )-1} \,\text { and }\, \lambda \Big (-\frac{1}{\tau }\Big ) = 1 - \lambda (\tau ), $$

which are FunGrim [19] properties bbfb6c and 07bf27, respectively.

Owing to the fact that $\lambda (\tau )$ is analytic on $\mathbb H$, all other poles of $h(\tau )$ are images of $\tau _h$ under the action of matrices

$$ \left( \begin{matrix} a& b\\ c& d \end{matrix} \right) \in \Gamma =\bigg \langle \Gamma (2), \left( \begin{matrix} 1& 0\\ 1& 1 \end{matrix} \right) , \left( \begin{matrix} 0& -2\\ 1& 0 \end{matrix} \right) \bigg \rangle . $$

All these images have imaginary part $\le 1$ because of

$$ \Im \Big ( \left( \begin{matrix} a& b\\ c& d \end{matrix} \right) \tau _h \Big ) =\frac{a d - b c}{|c \tau _h + d|^2}\cdot \Im (\tau _h) =\frac{a d - b c}{(c+d)^2+c^2}\cdot \Im (\tau _h) \le \Im (\tau _h) = 1. $$

This proves (129). $$\square $$

Another observation concerns a criterion for $|256 h(\tau )|<1$ to make the series in (127) converge:

### Lemma 11.1

Let

130 $$\begin{aligned} u:=5 + 4 \sqrt{2} - 2 \sqrt{2 (7 + 5 \sqrt{2})} = 0.0470219\dots \end{aligned}$$

and

$$ l:=5 + 4 \sqrt{2} + 2 \sqrt{2 (7 + 5 \sqrt{2})} = 21.26668\dots \,. $$

Then for all $\tau \in \mathbb H$ such that

131 $$\begin{aligned} |\lambda (\tau )| < u \,\text { or }\, |\lambda (\tau )|> l, \end{aligned}$$

one has

132 $$\begin{aligned} |256\ h(\tau )|< 1. \end{aligned}$$

### Proof of Lemma 11.1

Let $\Im (\tau )>1$ and $M:=|\lambda (\tau )|$, then

$$ |256\ h(\tau )| =\Big | 16\frac{\lambda (\tau ) (1 - \lambda (\tau ))^2}{ (1 + \lambda (\tau ) )^4} \Big | \le 16 \frac{M (1 + M)^2}{(1 - M)^4}. $$

We want,

$$ 16 \frac{M (1 + M)^2}{(1 - M)^4} < 1. $$

One has,

$$ 16\,M (1 + M)^2 - (1 - M)^4 = p_1 (-p_2), $$

where

$$ p_1 = M^2-2(5+4\sqrt{2}) M +1 \,\text { and }\, p_2 = M^2-2(5-4\sqrt{2}) M +1. $$

Now, in view of the simple facts,

$$ -p_2\le -1 \,\text { for }\, M> 0, \,\text { and }\, p_1=(M-u)(M-l), $$

one has for $M>0$,

$$ 16 \frac{M (1 + M)^2}{(1 - M)^4}< 1 \,\text { iff }\, p_1 (-p_2)<0 \,\text { iff }\, p_1>0 \,\text { iff }\, (M<u \,\text { or }\, M>l). $$

This completes the proof of Lemma 11.1. $\square$

Finally, in view of Lemma 11.1, we obtain a desired bound $L\ge 1$ by choosing *L* such that

133 $$\begin{aligned} |\lambda (\tau )| < u \,\text { for all }\, \tau \in \mathbb H\,\text { with }\, \Im (\tau )>L. \end{aligned}$$

Indeed, it turns out that such *L* exists.

### Proposition 11.2

For $L=1.87$ the upper bound *u* as in (133) holds.

### Proof

By (126) the inequality for *u* in (133) is equivalent to

134 $$\begin{aligned} |\theta _2(\tau )|< u^{1/4} |\theta _3(\tau )|, \end{aligned}$$

where $u^{1/4}$ denotes the real fourth root; i.e.,

$$ u^{1/4} = 0.46566654962266\dots \,. $$

By (124),

$$ |\theta _2(\tau )| =\left| 2x^{1/4}\sum _{n=0}^{\infty }x^{n^2+n}\right|<2|x|^{1/4}\sum _{n=0}^{\infty }|x|^{n^2+n} <2|x|^{1/4}\sum _{k=0}^{\infty }|x|^k=\frac{2|x|^{1/4}}{1-|x|}. $$

By (125),

$$\begin{aligned} |\theta _3(\tau )|&=\left| 1+2\sum _{n=1}^{\infty }x^{n^2}\right|>|1+2x|-2\left| \sum _{n=2}^{\infty }x^{n^2}\right|> |1+2x|-2\sum _{n=2}^{\infty }|x|^{n^2}\\&>|1+2x|-2|x|^4\sum _{k=0}^{\infty }|x|^k =|1+2x|-\frac{2|x|^4}{1-|x|} >1-2|x|-\frac{2|x|^4}{1-|x|} \end{aligned}$$

As a consequence, to guarantee (134) and thus (133), it suffices to choose $L\ge 1$ such that for all $x=e^{\pi i \tau }$ where $\tau = a + i b$ with $b>L$,

135 $$\begin{aligned} \frac{2|x|^{1/4}}{1-|x|} <u^{1/4} \left( 1-2|x|-\frac{2|x|^4}{1-|x|}\right) . \end{aligned}$$

In view of

$$ X:=|x|=|e^{\pi i (a + i b)}| = e^{- \pi b} < e^{- \pi L} \le e^{- \pi }=0.04321\dots \, , $$

we can restrict to assume

136 $$\begin{aligned} 0<X=|x|<27/625=0.0432<e^{-\pi }. \end{aligned}$$

To determine the real solutions $X:=|x|$ to the inequality (135) we run Mathematica’s cylindrical algebraic decomposition (CAD) procedure: 
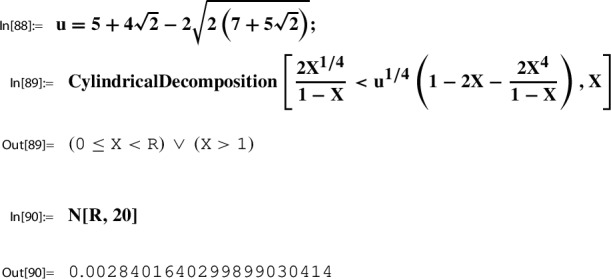
 Because of the constraint (136), the only remaining solution interval is

$$ 0< X< R. $$

Mathematica allows to determine the upper bound *R* with arbitrary precision; in Out[90] we computed 20 digits.

Summarizing, all $\tau =a + i b$ with real *a* and $b>0$ such that $e^{-\pi b}<R$ satisfy the inequality (133). Observing that

$$ b=-\frac{1}{\pi } \ln R = 1.86653526348736587369\dots $$

confirms that one can choose

$$ L=1.87 $$

to guarantee (133). This completes the proof of Proposition 11.2, and thus also (127) is proven. $\square$

### Remark 11.3

Mathematica represents the upper bound *R* with the data

137 $$\begin{aligned} \textrm{Root}[\{P_1,P_2\},\{3,1\}] \end{aligned}$$

where

$$ P_1=P_1(z_1)=1 - 4 z_1^2 - 2 z_1^4 - 4 z_1^6 + z_1^8 $$

and

$$ P_2=P_2(z_1,z_2) = z_1^4 - 16 z_2 - 12 z_1^4 z_2 + 62 z_1^4 z_2^2 -\dots + 16 z_1^4 z_2^{16}. $$

The data (137) specifies *R* as follows. By inspection one sees that $P_1(z_1)$ has four real roots which are ordered according to size as $r_1<r_2<r_3<r_4$. The 3 in the pair $\{3,1\}$ of (137) tells us to take $r_3$ and to solve $P_2(r_3,z_2)=0$ for $z_2$. This equation has two real solutions $s_1$ and $s_2$ such that $s_1<s_2$. Finally, the 1 in the pair $\{3,1\}$ of (137) tells us to take $s_1$ to obtain *R*; i.e.,

$$ R=s_1=\,\text { smallest real root of }\, P_2(r_3,z_2). $$

## Proof of Lemma 3.14: Modularity of *g* and *h*

Recall

$$ \Gamma :=\langle \Gamma (2), \gamma _1,\gamma _2 \rangle \,\text { with }\, \gamma _1:=\left( \begin{matrix} 1& 0\\ 1& 1 \end{matrix} \right) \,\text { and }\, \gamma _2:=\left( \begin{matrix} 0& -2\\ 1& 0 \end{matrix} \right) . $$

### Proof of $g\in M_2(\Gamma ; \chi )$

Let

$$ g(\tau ):=(1+\lambda (\tau ))\theta _3(\tau )^4. $$

We will show that

138 $$\begin{aligned} g(\gamma \tau ) = \chi (\gamma ) \det (\gamma )^{-1} (c \tau +d)^2 g(\tau ) \,\text { for all }\, \gamma \in \langle \Gamma (2), \gamma _1,\gamma _2 \rangle , \end{aligned}$$

where

$$ \chi (\gamma _1)=1, \chi (\gamma _2)=-1, \,\text { and }\, \chi (\gamma )=1 \,\text { for all }\gamma \in \Gamma (2), $$

This means, to prove (40),

$$ g\in M_2(\Gamma ; \chi ), $$

it is sufficient to prove (138) for the additional generators $\gamma _1$ and $\gamma _2$. To this end, we will use properties listed in the FunGrim library at www.fungrim.org.

FunGrim property 099301 for $\lambda$ gives,

139 $$\begin{aligned} \lambda (\gamma _1 \tau )=\frac{1}{\lambda (\tau )}. \end{aligned}$$

FunGrim property 4d8b0f for $\theta _j(z,\tau )$ gives,

140 $$\begin{aligned} \theta _3(\gamma _1 \tau )^4=(\tau +1)^2 \theta _2(\tau )^4. \end{aligned}$$

Consequently,

141 $$\begin{aligned} g(\gamma _1 \tau )&= (1+\lambda (\gamma _1 \tau ))\theta _3(\gamma _1 \tau )^4 = \left( 1+\frac{1}{\lambda (\tau )} \right) (\tau +1)^2 \theta _2(\tau )^4 \nonumber \\&= (\tau +1)^2 \left( 1+\frac{\theta _3(\tau )^4}{\theta _2(\tau )^4} \right) \theta _2(\tau )^4 = (\tau +1)^2 (1+\lambda (\tau ))\theta _3(\tau )^4 \nonumber \\&= (\tau +1)^2 g(\tau ). \end{aligned}$$

With regard to $\gamma _2$ the proof is as follows,

142 $$\begin{aligned} \lambda (\gamma _2 \tau )=\lambda \left( \frac{-2}{\tau }\right) =\lambda \left( -\frac{1}{\tau /2}\right) =\lambda \left( \left( \begin{matrix} 0& -1\\ 1& 0 \end{matrix} \right) \frac{\tau }{2} \right) = 1 -\lambda \left( \frac{\tau }{2} \right) , \end{aligned}$$

where the last equality is by FunGrim property 07bf27.

FunGrim property c4b16c for $\theta _3(z,\tau )$ gives,

143 $$\begin{aligned} \theta _3(\gamma _2 \tau )^4 =-\left( \frac{\tau }{2}\right) ^2 \theta _3 \left( \frac{\tau }{2} \right) ^4. \end{aligned}$$

Consequently,

$$\begin{aligned} g(\gamma _2 \tau )&= (1+\lambda (\gamma _2 \tau ))\theta _3(\gamma _2 \tau )^4 =- \left( \frac{\tau }{2}\right) ^2 \left( 2 -\lambda \left( \frac{\tau }{2} \right) \right) \theta _3 \left( \frac{\tau }{2} \right) ^4. \end{aligned}$$

To complete the proof we will show that

144 $$\begin{aligned} g(\gamma _2 \tau )=- \frac{1}{\det (\gamma _2)} \tau ^2 g(\tau ), \end{aligned}$$

which is equivalent to showing

$$ \left( 2 -\lambda \left( \frac{\tau }{2} \right) \right) \theta _3 \left( \frac{\tau }{2} \right) ^4 = 2 (1+\lambda (\tau ))\theta _3(\tau )^4. $$

Using the definition of $\lambda$ in terms of theta functions, we rewrite this as

$$ 2 \theta _3 \left( \frac{\tau }{2}\right) ^4 - \theta _2 \left( \frac{\tau }{2}\right) ^4 = 2 \theta _3(\tau )^4 + 2 \theta _2(\tau )^4. $$

Applying FunGrim property 69b32e for $\theta _3(z,\tau /2)$ this equality turns into

$$ 2\left( \theta _3(\tau )^2 + \theta _2(\tau )^2 \right) ^2 - \theta _2 \left( \frac{\tau }{2}\right) ^4 =2 \theta _3(\tau )^4 + 2 \theta _2(\tau )^4, $$

which reduces to

$$ 4 \theta _2(\tau )^2 \theta _3(\tau )^2 =\theta _2 \left( \frac{\tau }{2}\right) ^4. $$

This equality can be derived by using FunGrim properties for $\theta _j(0,\tau )$ again:

$$\begin{aligned} 4 \theta _2(\tau )^2 \theta _3(\tau )^2&= 4^2 \frac{\eta (\tau )^6}{\theta _4(\tau )^2} \hspace{0.6cm}\text {(by FunGrim 557b19)}\\&= 4^2 \frac{\eta (\tau )^8}{\eta (\tau /2)^4} \hspace{0.4cm}\text {(by FunGrim 9448f2)}\\&= \theta _2 \left( \frac{\tau }{2}\right) ^4 \hspace{0.4cm}\text {(by a9c825).} \end{aligned}$$

This completes the proof of (144), and by combining this with (141), also (40) and (138) are proven.

### Proof of $h\in M_0(\Gamma )$

Let

$$ h(\tau ):= \frac{ \lambda (\tau ) (1-\lambda (\tau ))^2}{16 (1+\lambda (\tau ))^4}. $$

We will show that

145 $$\begin{aligned} h(\gamma \tau ) = h(\tau ) \,\text { for all }\, \gamma \in \langle \Gamma (2), \gamma _1,\gamma _2 \rangle . \end{aligned}$$

The invariance property (145) for $\gamma \in \Gamma (2)$ is implied by the classical $\Gamma (2)$-invariance of $\lambda$. Consequently, to prove the remaining part of (52),

$$ h\in M_0(\Gamma ), $$

it is sufficient to prove the modular transformation property (145) for the extra generators $\gamma _1$ and $\gamma _2$.

Applying (139) to the definition of *h* gives,

$$ h(\gamma _1 \tau ) = \frac{ \lambda (\tau )^{-1} (1-\lambda (\tau )^{-1})^{2}}{16 (1+\lambda (\tau )^{-1})^{4}} = h(\tau ). $$

Applying (142) gives,

$$ h(\gamma _2 \tau ) = \frac{ \left( 1 -\lambda \left( \frac{\tau }{2} \right) \right) \lambda \left( \frac{\tau }{2} \right) ^2}{16\left( 2 -\lambda \left( \frac{\tau }{2} \right) \right) ^4}; $$

i.e., to show $h(\gamma _2 \tau )=h(\tau )$ is equivalent to proving that for $z=\lambda (\tau )$ and $y=\lambda (2\tau )$,

$$\begin{aligned} 0&=\frac{(1-z)z^2}{(2-z)^4} - \frac{y(1-y)^2}{(1+y)^4}\\&=\frac{(1-z-y) (1+z y-y) \left( z^2 y^2-2 z^2 y+z^2+16 z y-16 y\right) }{(2-z)^4 (y+1)^4}. \end{aligned}$$

The verification of the respective modular equation of level 2:

146 $$\begin{aligned} \lambda (\tau )^2 \lambda (2\tau )^2-2 \lambda (\tau )^2 \lambda (2\tau )+\lambda (\tau )^2+16 \lambda (\tau ) \lambda (2\tau )-16 \lambda (2\tau )=0, \, \tau \in \mathbb H, \end{aligned}$$

can be done (algorithmically) by classical methods which completes the proof of $h\in M_0(\Gamma )$.

#### Remark 12.1

The modular equation (146) can be found and proven algorithmically; see the next Sect. 12.3.

### Finding and proving (146) algorithmically

The algorithmic discovery of (146) can be done as follows. Taking as input the first 15 terms of $y:=\lambda (2 \tau )=\sum _{n=1}^\infty a(n) x^n$ and $z:=\lambda (\tau )=\sum _{n=1}^\infty b(n) x^n$, $x=\exp (\pi i \tau )$, will be sufficient:
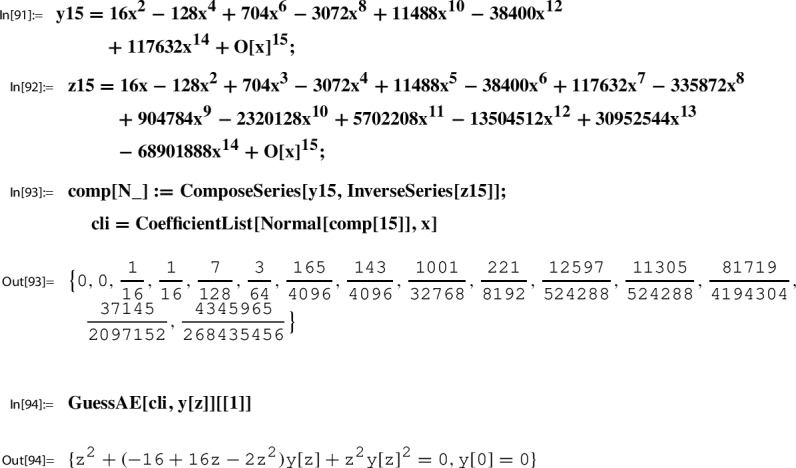
 This output means that

$$\begin{aligned} 0&= \lambda (\tau )^2 + \left( -16 + 16 \lambda (\tau ) - 2 \lambda (\tau )^2\right) \lambda (2\tau ) + \lambda (\tau )^2 \lambda (2\tau )^2\\&= \lambda (\tau )^2 \lambda (2\tau )^2-2 \lambda (\tau )^2 \lambda (2\tau )+\lambda (\tau )^2+16 \lambda (\tau ) \lambda (2\tau )-16 \lambda (2\tau ), \end{aligned}$$

where the last line is nothing but (146).

The proof of this algorithmic discovery of (146) can be done using the algorithm ModFormDE as described in [20] and [21]. The basis for this kind of proof is the differential equation obtained by Alternatively, one can verify (146) by checking the local expansions at the cups; an example of this method can be found in Sect. 7.1.

## Conclusion

In the Introduction we mentioned Zudilin’s article [32] entitled “Ramanujan-type formulae for $1/\pi$: A second wind?” and expressed our hope that the methods presented in this article will bring new “algorithmic wind” into this area. We want to stress that our hope is not restricted to the topic discussed in this paper, but also extends to a variety of other possible areas where the algorithm MultiSamba, or its interplay with holonomic methods and the algorithm ModFormDE, may provide new possibilites for research.

## Data Availability

No datasets were generated or analysed during the current study.

## Footnotes

1 E.g., in [11, p. 45] they state, “Expression (99) can be used whenever $\tau =(1+\sqrt{-d})/2$.”

2 E.g., as here, to exploit sparsity of terms.
